# psupertime: supervised pseudotime analysis for time-series single-cell RNA-seq data

**DOI:** 10.1093/bioinformatics/btac227

**Published:** 2022-06-27

**Authors:** Will Macnair, Revant Gupta, Manfred Claassen

**Affiliations:** Institute of Molecular Systems Biology, Department of Biology, ETH Zurich, Zurich 8093, Switzerland; Inner Medicine I, Faculty of Medicine, University of Tübingen, University Hospital Tübingen, 72074, Germany; Inner Medicine I, Faculty of Medicine, University of Tübingen, University Hospital Tübingen, 72074, Germany; Department of Computer Science, University of Tübingen, Tübingen 72074, Germany

## Abstract

**Motivation:**

Improvements in single-cell RNA-seq technologies mean that studies measuring multiple experimental conditions, such as time series, have become more common. At present, few computational methods exist to infer time series-specific transcriptome changes, and such studies have therefore typically used unsupervised pseudotime methods. While these methods identify cell subpopulations and the transitions between them, they are not appropriate for identifying the genes that vary coherently along the time series. In addition, the orderings they estimate are based only on the major sources of variation in the data, which may not correspond to the processes related to the time labels.

**Results:**

We introduce psupertime, a supervised pseudotime approach based on a regression model, which explicitly uses time-series labels as input. It identifies genes that vary coherently along a time series, in addition to pseudotime values for individual cells, and a classifier that can be used to estimate labels for new data with unknown or differing labels. We show that psupertime outperforms benchmark classifiers in terms of identifying time-varying genes and provides better individual cell orderings than popular unsupervised pseudotime techniques. psupertime is applicable to any single-cell RNA-seq dataset with sequential labels (e.g. principally time series but also drug dosage and disease progression), derived from either experimental design and provides a fast, interpretable tool for targeted identification of genes varying along with specific biological processes.

**Availability and implementation:**

R package available at github.com/wmacnair/psupertime and code for results reproduction at github.com/wmacnair/psupplementary.

**Supplementary information:**

Supplementary data are available at *Bioinformatics* online.

## 1 Introduction

**Fig. 1.**
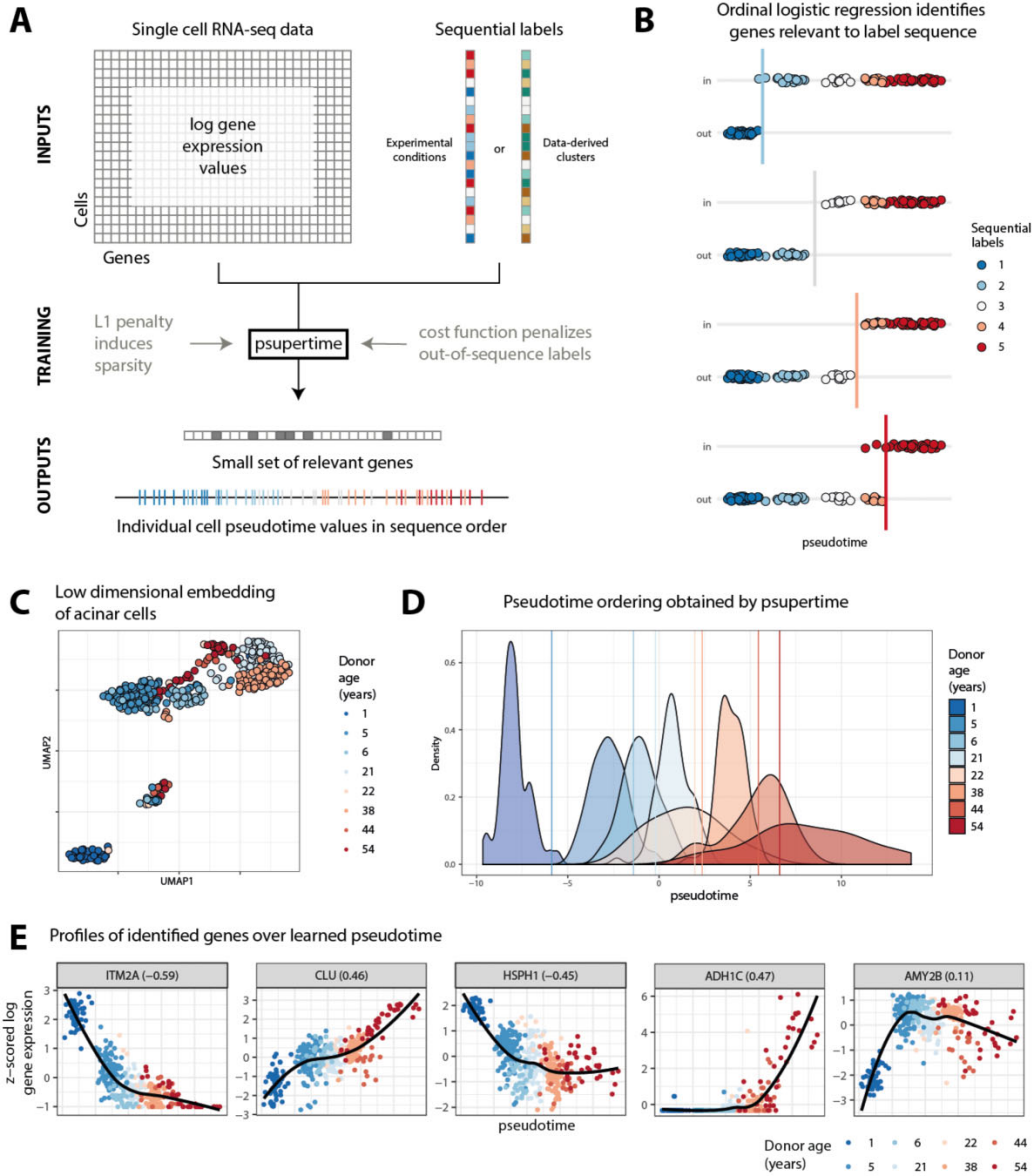
(**A**) Inputs to psupertime are single-cell RNA-seq data, where the cells have sequential labels associated with them. psupertime then identifies a sparse set of ordering coefficients for the genes. Multiplying the gene expression values by this vector of coefficients gives pseudotime values for each cell, which place the labels approximately in sequence. (**B**) Cartoon of statistical model used by psupertime, including thresholds between labels. Where there is a sequence of *K* condition labels, psupertime learns *K−*1 simultaneous (i.e. sharing coefficients) logistic regressions, each seeking to separate labels 1…k−1 (out) from k…K (in). (**C**) Dimensionality reduction of 411 human acinar cell data with ages ranging from 1 to 54 (Enge *et al.*, 2017). Representations in two dimensions via non-linear dimensionality reduction technique UMAP. Colours indicate donor age. (**D**) Distributions of donor ages for acinar cells over the pseudotime learned psupertime. Vertical lines indicate thresholds learned by psupertime distinguishing between earlier and later sets of labels; colour corresponds to the next later label. (**E**) Expression values of selected genes (five with largest absolute coefficients; see Supplementary Fig. S2 for 20 largest). The *x*-axis is psupertime value learned for each cell; *y*-axis is z-scored ${\;\text{log}\;}_{2}$ gene expression values. Gene labels also show the Kendall’s *τ* correlation between sequential labels (treated as a sequence of integers 1,…,K) and gene expression

Single-cell RNA-sequencing studies have been used to define the transcriptional changes in biological time series, including embryonic development (Petropoulos *et al.*, 2016), response to stimulus (Treutlein *et al.*, 2016), differentiation (Bendall *et al.*, 2014) and ageing (Enge *et al.*, 2017). Such studies are based on single-cell RNA-seq measurements over a sequence of experimental labels of the successive timepoints. These data are typically analysed using unsupervised pseudotime techniques to extract the corresponding temporal sequence of transcriptomic states. These approaches use similarities between cells to computationally order them along trajectories, allowing researchers to identify high-level cell subpopulations and the transitions between them. However, unsupervised methods are not designed to identify genes associated with a process unfolding over time. In addition, they assume that the major driver of variation in the data is most indicative of the time series-induced cell orderings. This means that where the changes along the time series are subtle, or where there are strong additional sources of variation, the orderings they identify may not be those associated with the time series (Saelens *et al.*, 2019). Only recently, approaches have been published to derive or refine pseudotime with time-series information (Shao *et al.*, 2021; Tran and Bader, 2020). To further address this methodological gap, we introduce a supervised pseudotime technique, psupertime, which explicitly uses time-series labels as input (Fig. 1A). psupertime is based on penalized ordinal regression (Fig. 1B), a statistical technique used where data have categorical labels that follow a sequence. psupertime produces three outputs. Firstly, it learns a small, interpretable set of genes that vary coherently over the time series. Secondly, a linear combination of these genes assigns a pseudotime value to each cell, which approximately recapitulates the ordering specified by the sequence of labels. Thirdly, it can be used to classify new data according to the process labelled in the data used for training. These outputs allow for targeted characterization of processes for any single-cell RNA-seq data where sequential labels are available (such as time, disease progression or unidimensional spatial measurements), despite substantial variation not associated with the process of interest. Full details of the method are given in Section 2.

We demonstrate psupertime on a dataset comprising 411 acinar cells from the pancreas, from eight human donors with ages from 1 to 54 years (Enge *et al.*, 2017). Acinar cells perform the exocrine function of the pancreas, producing enzymes for the digestive system. This dataset was selected because each set of cells was obtained from different donors, resulting in significant variation in the dataset unrelated to donor age (Fig. 1C). Despite this variation, psupertime finds a cell-level ordering which respects the age progression, while separating the labels from each other (Fig. 1D). We show that the performance of psupertime is robust, including perturbations in labels (see Supplementary Results S1).

## 2 Materials and methods

### 2.1 Overview of psupertime methodology

psupertime requires two inputs: (i) a matrix of log read counts from single-cell RNA-seq, where columns correspond to genes and rows correspond to cells; and (ii) a set of labels for the cells, with a defined sequence for the labels (e.g. a set of cells could have labels *day1*, *day3*, *day1*, *day2*, *day3*). (Note that not all cells need to be labelled: psupertime can also be run on a labelled subset.) psupertime then identifies a set of ordering coefficients, *β_i_*, one for each gene (Fig. 1A). Multiplication by this vector of coefficients converts the matrix of log gene expression values into pseudotime values for each individual cell. The set of pseudotime values recapitulates the known label sequence (so the cells with labels *day1* will on average have lower pseudotime values than those labelled *day2* and so on). The vector of coefficients is *sparse*, in the sense that many of the values are zero; these therefore have no influence on the ordering of the cells. Genes with non-zero coefficients are therefore identified by psupertime as relevant to the process which generated the sequential labels.

Suppose the sequence of condition labels we have is 1,…,K. Intuitively, psupertime learns a weighted average of gene expression values that separates the cells with label 1 from the cells with labels 2,…,K, at the same time as separating 1, 2 from 3,…,K, and 1, 2, 3 from 4,…,K and so on (Fig. 1B). This can be thought of as solving *K—*1 simultaneous logistic regression problems and is termed *ordinal logistic regression* (McCullagh, 1980).

As described so far, psupertime can be thought of as minimizing a cost, where the cost is the error in the resulting ordering. To make the results more interpretable, we would like psupertime to use a small set of genes for prediction. To do this, we add a cost for each coefficient *β_i_* used, so that psupertime is minimizing $\mathrm{error}+\lambda \sum_{i}|\beta_{i}|$; approaches like this are termed *regularization*, and in this case *L1 regularization*. The parameter *λ* controls the balance between minimizing error, and minimizing the ‘coefficient cost’. The method for implementing this approach is based on the R package glmnetcr, which we have extended with an additional statistical model.

The results of this procedure are: (i) a small and therefore interpretable set of genes with non-zero coefficients; (ii) a pseudotime value for each individual cell, obtained by multiplying the log gene expression values by the vector of coefficients; and (iii) a set of values along the pseudotime axis indicating the thresholds between successive sequential labels (these can then be used for classification of new samples). Where the data do not have condition labels, psupertime can be combined with unsupervised clustering to identify relevant processes (see Supplementary Results S3).

### 2.2 Pre-processing of data

To restrict the analysis to relevant genes and denoise the data, psupertime first applies pre-processing to the log transcripts per million values. Specifically, psupertime first restricts to highly variable genes, as defined in the scran package in R, i.e. genes that show above the expected variance relative to genes with similar mean expression (Lun *et al.*, 2016). Genes that are only expressed in a small number of cells (the default is 1%) are excluded. psupertime implement data denoising and dropout correction by calculating correlations between the log expression values across all selected genes for each pair of cells, using the correlations to identify the 10 nearest neighbours for each cell and replacing the value for a given cell by the mean value over these neighbours. Finally, the resulting log-count values for each gene are scaled to have mean zero and standard deviation one.

### 2.3 Penalized ordinal logistic regression

psupertime applies cross-validated regularized ordinal logistic regression to the processed data, using the labels as the sequence. Ordinal logistic regression is an extension of binary logistic regression to an outcome variable with more than two labels, where the labels have a known or hypothesized sequence. The likelihood for ordinal logistic regression is defined by multiple simultaneous logistic regressions, where each one models the probability of a given observation having an earlier or later label, with the definition of ‘early’/‘late’ differing across the simultaneous regressions (Fig. 1B). The same linear combination of input variables is used across all individual logistic regressions. This specific model of ordinal logistic regression, in which the simultaneous logistic regressions each seek to separate labels 1…k from labels k+ 1…K, is termed *proportional odds*. (A commonly used alternative is the *continuation ratio* model, where the regressions seek to separate labels 1…k from label *k *+* *1 alone. This is also implemented as an option in psupertime.)

In the case where the number of input variables is high relative to number of observations and may include many uninformative variables, as is common in single-cell RNA-seq, it can be helpful to introduce sparsity (i.e. to increase the number of zero coefficients). psupertime uses *L1 regularization* to do this. Our approach is based on that in the R package glmnetcr (Archer and Williams, 2012), which reformulates the data and associated likelihood functions into one single regression model, to take advantage of the fast performance of the glmnet package (Friedman *et al.*, 2010). The model originally implemented in glmnetcr is the continuation ratio likelihood; we have extended this approach to implement the proportional odds likelihood, as this model is more appropriate for assessing an entire biological process. Under the proportional odds assumption, the two categories are: categories *j* and higher, and categories lower than *j*; the regression therefore estimates log(P(Y>=j)/P(Y<j)). Under the continuation ratio assumption, the two categories are: *j*, and categories lower than *j*; here, the regression estimates log(P(Y=j)/P(Y<j)). Intuitively, the proportional odds framework models an observation’s global progression along the ordinal values, while the continuation ratio framework models the probability of proceeding to the next ordinal value. For most of the examples that we have seen, such as studying development or ageing, the proportional odds framework is appropriate. However, the continuation ratio framework may be appropriate in some cases, for example in disease progression, or evolutionary processes. Given input data $X\in R^{n\times p}$ and $y\in N^{n}$ condition labels (which for simplicity we assume are integers), this results in the following cumulative distribution function for ordinal logistic regression:

$$P(y_{i}\leq j|X_{i})=\phi (\theta_{j}-\beta^{T}X_{i})=\frac{1}{1+\text{exp}(\beta^{T}X_{i}-\theta_{j})}.$$

Here, *X_i_* and *y_i_* are the vector and integer corresponding to the *i*th observation and label respectively, *j* indicates one of the possible condition labels, *β* is the vector of coefficients and $\left\{\theta_{j}\right\}$ are the thresholds between labels. ϕ is the logit link function, which transforms the linear combination of predictors into a probability. Note that the probability given here is cumulative and that to calculate the probability of an individual label, we have to calculate the difference between successive labels. This results in the following *unpenalized* likelihood:

$$L(\beta ,\theta |y,X)=\prod_{i=1}^{N}(\phi (\theta_{y_{i}}-\beta^{T}X_{i})-\phi (\theta_{y_{i}-1}-\beta^{T}X_{i})),$$

where *y_i_* is the label of observation *i*. Including the L1 penalty, for a given value of *λ*, we obtain the optimal values of *β* and *θ* by maximizing the following penalized objective function:

$$\underset{\beta ,\theta }{\text{argmax}}(\text{log}\;L(\beta ,\theta |y,X)-\lambda \sum_{i=1}^{p}|\beta_{i}|).$$

psupertime uses cross-validation (with 5 folds as default) to identify the optimal level of L1 regularization: the optimal *λ* is the value with the highest mean score over all held-out folds (either accuracy or cross-entropy may be selected as the score; the default is cross-entropy). To increase sparsity, we use the highest value of *λ* with mean training score within one standard error of the optimal *λ*, rather than take the optimal *λ* itself [following Friedman *et al.* (2010)]. The model is then retrained using all training data, with this value of *λ*, to obtain the best-fitting model.

Where psupertime is used to classify completely new data (e.g. from a different experiment), to make the predictions more robust, the cross-validation should take data structure into account (e.g. selecting entire samples to be left out, rather than cells selected at random).

### 2.4 Psupertime outputs

The psupertime procedure results in a set of coefficients for all input genes (many of which will be zero) that can be used to project each cell onto a pseudotime axis, and a set of cut-offs indicating the thresholds between successive sequential labels (Fig. 1D). These can be analysed in various useful ways.

The small, interpretable set of genes reported to have non-zero coefficients permits both validation that the procedure has been successful (by observation of genes known to be relevant to the process) and discovery of new relevant genes. The magnitude of a coefficient is a measure of the contribution of this gene to the cell ordering. More precisely, for a gene *i* with coefficient *β_i_*, each unit increase in log transcript abundance multiplies the odds ratio between earlier and later labels by $e^{\beta_{i}}$. Where *β_i_* is small, a Taylor expansion indicates this is approximately equal to a linear increase by a factor of *β_i_*.

The thresholds indicate the points along the psupertime axis at which the probability of label membership is equal for labels before the cut-off, and after the cut-off. The distances between thresholds, namely the size of transcriptional difference between successive labels, are not assumed to be constant and are learned by psupertime. Distances between thresholds therefore indicate dissimilarity between adjacent labels, and thresholds which are close together suggest labels which are transcriptionally difficult to distinguish.

The learned geneset can also be used as input to dimensionality reduction algorithms such as t-SNE or UMAP; this is discussed in more detail in Supplementary Results S4.

Rather than learning a pseudotime for one fixed set of input points, psupertime learns a function from transcript abundances to the pseudotime. It can therefore be trained on one set of labels and applied to new data with unknown or different labels: any data with overlapping gene measurements can be assessed with regard to the learned process. Furthermore, psupertime can be learned on two different datasets, with different labels, and then each applied to the other dataset: the sequential labels from one dataset allow coefficients relevant to that sequence to be learned, which can then be used to predict these labels for the second dataset. See Supplementary Figure S23 for more discussion.

### 2.5 Simulations of single-cell RNA-seq data

psupertime is principally useful because it can identify genes which vary over the course of time-series labels. To test this capability, we simulated single-cell RNA-seq data to include three types of gene profiles, defined in terms of their mean expression: mean varying as a time series; sample-specific variation in the mean; and constant mean expression. All genes have biological and technical noise around this mean. This mimics the likely experimental setup, in which the expression at each timepoint is composed of both processes related to the time series, and unrelated variability particular to that sample, e.g. where the samples are derived from different mice.

Our simulation procedure was as follows: (i) calculate relevant statistics from a selected reference dataset, composed of multiple labels, (ii) randomly sample latent time values for each cell, around a common mean for the cell’s label, (iii) randomly assign one of the three gene profile types to each gene and randomly sample some parameters for each gene and (iv) sample counts for each cell and gene based on the combination of cell- and gene-level parameters. We discuss each of these steps in turn.

As a reference dataset, we used 575 mouse embryonic beta cells (Qiu *et al.*, 2017a), restricted to 2666 highly variable genes by the procedure described in Lun *et al.*, where the cells were labelled with seven distinct time labels. The statistics used were library size for each cell (i.e. the total number of reads observed) and the mean *μ_g_* and dispersion *ρ_g_* for each gene *g* (calculated using edgeR; Robinson *et al.*, 2010), assuming a negative binomial distribution. In each simulation, the library sizes of cells were randomly permuted, and the number of cells allocated to each label was randomly permuted.

To sample the latent time values for label *i*, *l_i_*, we assumed an exponential distribution of time until the next timepoint. The first time point label has mean value 0, then the time to each subsequent timepoint is drawn from an exponential distribution with rate 0.5 (i.e. mean time difference of 2): $l_{i}|l_{i-1}∼\;\text{Exp}(0.5)+l_{i-1}$. To allow for cell-to-cell variability, we then add Gaussian noise to the values for each cell *c*, with mean 0 and standard deviation 1: $t_{c}|l_{i}∼N(l_{i},1)$. This results in a latent time value for each cell. We then scale these values to have minimum 0 and maximum 1.

The three possible types of gene expression profile that we defined were: time series; label-specific; and non-specific. Each gene follows one of these profiles. Each gene has dispersion and base mean expression defined by the reference dataset. The gene expression profiles were simulated as follows:

- Time-series genes have expression which changes with respect to the latent time values for each cell, where the log fold change relative to the mean follows a logistic curve. This curve is defined by three values: *t*_0_, the curve’s midpoint; *k*, half the derivative of the curve at that midpoint; and *L*, the asymptotic maximum value of the curve. The log mean expression of this gene in a cell with latent time value *t_c_* is therefore $\text{log}(\mu_{g})+L*\text{logistic}((t_{c}-t_{0})*k)$. For each gene, we sampled *t*_0_ from a uniform distribution over [0,1]; *k* from a log10-normal distribution with mean 1 and standard deviation 1; and *L* from a gamma distribution with shape 4 and rate 2.
- Label-specific gene profiles are defined by two parameters: the sample in which they show differential expression, and the log fold change in that sample relative to the mean. For each gene, we uniformly at random select a label, and sample the log fold change from a gamma distribution with shape 4 and rate 2.
- Genes with non-specific expression are defined by the dispersion and base mean identified from the reference dataset, and have no difference in distribution across labels.

Each simulation has a defined set of proportions for each type of gene profile, $\left(p_{\text{ts}},p_{\text{label}},p_{\text{non}}\right)$. Each gene is randomly assigned one of the types according to these probabilities.

We now have all the parameters required to sample counts for each combination of cell and gene. The gene-level parameters define, via the combination of base mean expression and possibly also a log fold change relative to the base mean, the mean expression for a given gene, plus its dispersion. The cell-level parameters define the library size for each cell, which is used to scale the base mean. For each cell and gene combination, we sample from the defined negative binomial distribution.

### 2.6 Simulations of single-cell RNA-seq data with cell types

To simulate time-series data comprising multiple cell types, we used fluorescence-activated cell-sorted stem cells at different stages of differentiation (Koh *et al.*, 2016), which had previously been used for benchmarking (Duò *et al.*, 2018). We assumed that genes had the following four profile types: global time-series, cell-type time series, batch effect and non-specific genes. Global time-series genes have the same timing and effect size on gene expression for all cell types; cell-type time-series genes vary over time in all cell types, but the timing and strength of effect are variable between cell types. Specifically, we sampled the parameters defining the response to time series in exactly the same way as for our previous simulations (see Section 2.5); however, for globally varying genes, these parameters were constant across cell types, while for cell-type varying genes, these parameters were sampled independently for each cell type. After simulating count data, we applied psupertime to each cell type individually, and to all cell types grouped together.

### 2.7 Benchmarking of time-series gene identification against classifiers

psupertime is a classifier that identifies a small subset of relevant features. We therefore compared it to alternative classification methods, which also produce variable importance measures.

Multinomial regression is a simple baseline approach to classification (Venables and Ripley, 2002). For each label, a linear logistic regression is performed to distinguish label from non-label, resulting in *k* coefficients for each gene. To identify relevant features, for each gene, we calculate the sum of squares of the *k* coefficients; where a gene is relevant for classifying many labels, or strongly relevant for one label, it will have a large combined weight.

Random forest is a widely used classification algorithm that is known to have good performance in many circumstances (Caruana *et al.*, 2008). One of the outputs produced by the algorithm is ‘importance’, which is the (relative) mean increase in error when a given variable is permuted. This can be used to identify which genes are most critical to classification performance.

To assess performance, we simulated single-cell RNA-seq data (as described in Section 2.5), assuming that mean gene expression followed one of three possible profiles: time-series, label-specific or a constant mean across labels. We varied the proportions of these types of gene, so that the proportion of genes following time-series profiles, $p_{\text{ts}}$, was 0.1, 0.3, 0.5, 0.7 or 0.9. The proportion of genes following label-specific profiles, $p_{\text{label}}$, was between 0.1 and $1-p_{\text{ts}}$. The proportion of non-specific genes, $p_{\text{non}}$, accounted for the remainder of genes.

For each triplet of distinct $\left(p_{\text{ts}},p_{\text{label}},p_{\text{non}}\right)$ values, we did 20 simulations starting from 20 different random seeds. For a given simulation, applying psupertime and the benchmark methods resulted in an ‘importance’ value for each gene. We used this variable to predict time-series-specific genes, and calculated precision-recall curves for each classifier on the basis of how successfully these values identified the true time-series genes.

We note that due to different combinations of randomly selected parameters, some time-series genes in the simulations will be easier to detect and some more difficult. For example, a gene with low absolute log fold change value *L*, and high dispersion *ρ*, will have a poor signal-to-noise ratio for the detection of time-series trends. This puts biologically realistic limits on the best performance possible for any algorithm, as for some genes any time-series trends will be obscured by transcriptional variability. For this reason, and also because psupertime is intended to identify a small set of genes, we have restricted our analysis to values of recall between 0% and 10%.

### 2.8 Benchmarking of cell orderings against pseudotime methods

Both psupertime and unsupervised pseudotime techniques produce a cell ordering, which may or may not correlate with the label ordering. We compared psupertime against unsupervised pseudotime methods, on five datasets with time-series labels (Table 1). We first performed common pre-processing and identification of relevant genes for each dataset, to identify either highly variable genes, or genes showing high correlation with the label sequence. See Supplementary Results S2 for further discussion.

**Table 1.** Details of datasets used in benchmarking of pseudotime cell orderings

Dataset name	Source	Accession	Labels used	No. of labels	No. of cells	No. of highly varying genes
Acinar cells	Enge *et al.* (2017)	GSE81547	Donor age	8	411	827
Human germline, F	Li *et al.* (2017)	GSE86146	Age (weeks)	12	992	1081
Embryonic beta cells	Qiu *et al.* (2017a)	GSE87375	Developmental stage	7	575	2666
Human ESCs	Petropoulos *et al.* (2016)	E-MTAB-3929	Embryonic day	5	1529	2876
MEF to neurons	Treutlein *et al.* (2016)	GSE67310	Days since induction	5	315	1698
Colon cells	Herring *et al*. (2017)	GSE102698	User-selected clusters	4, 5	1894	1515
iPSCs	Schiebinger *et al.* (2019)	GSE106340	Days during reprogramming	11	3600	731

To identify highly variable genes, we followed the procedure described by Lun *et al.*, using an false discovery rate (FDR) cut-off of 10% and biological variability cut-off of 0.5 [see Lun *et al.* (2016) for details of these parameters]. To identify genes showing high correlation with the labels, we calculated the Spearman’s correlation coefficient between sequential labels converted into integers, and log gene expression value. Genes with absolute correlation >0.2 were selected.

For principle component analysis (PCA), we calculated the first principal component of the log counts and used this as the pseudotime. Calculation of Monocle2 uses the following default settings: genes with mean expression <0.1 or expressed in <10 cells filtered out; negbinomial expression family used; dimensionality reduction method *DDRTree*; root state selected as the state with the highest number of cells from the first label; function orderCells used to extract the ordering.

Calculation of slingshot uses the following default settings: first 10 PCA components used as dimensionality reduction; clustering via Gaussian mixture model clustering using the R package mclust, number of clusters selected by Bayesian information criterion; root and leaf clusters selected as the clusters with highest number of cells from the earliest and latest labels, respectively; lineage selected for pseudotime is path from root to leaf cluster. *Note:* For cells very distant from the selected path, slingshot does not give a pseudotime value. For these cells, we assigned the mean pseudotime value over those that slingshot did calculate. Calculation of psupertime used default settings, as described in Section 2.

We tested the extent to which each pseudotime method could correctly order the cells by calculating measures of correlation between the learned pseudotime, and the sequential labels. Kendall’s *τ* considers pairs of points and calculates the proportion of pairs in which the rank ordering within the pair is the same across both possible rankings.

To identify genes with high correlation with the sequential condition labels (Supplementary Table S1), we treated the sequential labels as the set of integers 1,…,K, calculated the Spearman correlation coefficient with the gene expression. Genes were selected that showed absolute correlation of >0.2 with the sequential labels (few genes showed high correlation with the sequential labels; this low cut-off was used to ensure that a sufficient number of genes was selected).

### 2.9 Identification of relevant biological processes

To identify biological processes associated with the condition labels, psupertime first clusters all genes selected for training (e.g. the default highly variable genes), using the R package fastcluster, using five clusters by default. These are ordered by correlation of the mean expression values with the learned pseudotime, i.e. approximately into genes that are up- or down-regulated along the course of the labelled process. psupertime then uses topGO to identify biological processes enriched in each cluster, relative to the remaining clusters; enriched GO terms are calculated using algorithm = ‘weight’ and statistic = ‘fisher’ (Alexa and Rahnenführer, 2009).

## 3 Results

psupertime produces as output a set of ordering coefficients, one for each gene, most of which are zero (i.e. the coefficient vector is ‘sparse’). A non-zero ordering coefficient indicates that a gene was relevant to the label sequence. This balances the requirement for predictive accuracy against that for a small and therefore interpretable set of genes. For example, applied to the acinar cells, psupertime used 82 of the 827 highly variable genes to attain a test accuracy of 83% over the eight possible labels (Supplementary Fig. S1). Many of the genes identified via their absolute coefficient values are already known to be relevant to the ageing of pancreatic cells (see expression profiles shown in Fig. 1E, Supplementary Figs. S2 and S3). For example, clusterin (*CLU*) plays an essential role in pancreas regeneration and is expressed in chronic pancreatitis (Lee *et al.*, 2011; Xie *et al.*, 2002); *α*-amylase (*AMY2B*) is a characteristic gene for mature acinar cells, encoding a digestive enzyme (Omichi and Hase, 1993). In addition, psupertime suggests candidates for further study: *ITM2A* has the highest absolute gene coefficient and is highly differentially regulated in a model of chronic pancreatitis, but has not been investigated in acinar cells (Ulmasov *et al.*, 2013). The genes identified by psupertime were not discussed in the source manuscript, and, importantly, would not be found by naively calculating correlations between the sequential labels and gene expression (see Supplementary Results S2).

GO term enrichment analysis provides further support for the validity of the cell ordering identified by psupertime. We clustered the expression profiles of the highly variable genes and identified GO terms characteristic of each cluster (see Section 2). This procedure identified genes related to digestion as being up-regulated in early ages (‘proteolysis’ and ‘digestion’ enriched in cluster 1), and terms related to ageing later in the process (‘negative regulation of cell proliferation’ and ‘positive regulation of apoptotic process’ enriched in cluster 5; see Supplementary Figs. S4 and S5). This analysis confirms that the cell ordering learned by psupertime is plausible.

**Fig. 2.**
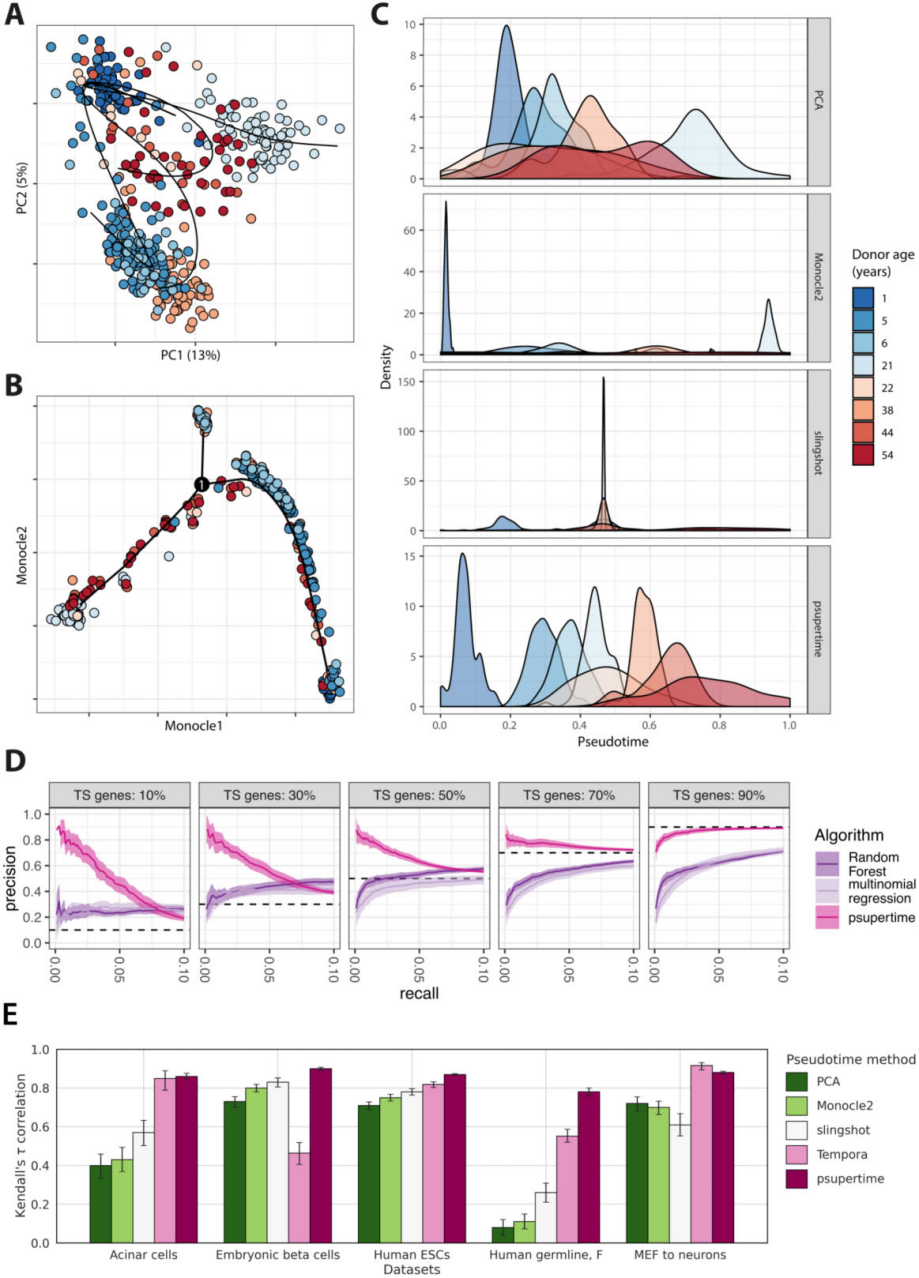
Performance of psupertime against benchmark methods. See Section 2.8 for details of data processing and use of benchmark methods. All results for (**A–C**) based on 411 aging human acinar cell data with ages ranging from 1 to 54 (Enge *et al.*, 2017), using 827 highly variable genes. Colours indicate donor age. (A) Projection of acinar cells into first two principal components (% of variance explained shown). Curves learned by slingshot shown (note that here we show the projection of these curves into the first two principal components). (B) Projection of acinar cells into dimensionality reduction calculated by Monocle 2, annotated with pseudotime learned by Monocle 2 (Qiu *et al.*, 2017b). (C) Results of benchmark pseudotime methods applied to acinar data. For each method, the *x*-axis is a one-dimensional representation for each cell (see Section 2.8), scaled to [0,1] and given the direction with the highest positive correlation with the label sequence. The *y*-axis is density of the distributions for each label used as input, as calculated by the function geom_density in the R package ggplot2. (**D**) Performance of psupertime and benchmark classifiers in identifying simulated time-series genes. Precision-recall curves based on identification of time-series genes via variable importance measures for each method (see Section 2.7). Line and area show mean and ±2 standard error, respectively, over 20 simulations. Recall is limited to range 0–10%. Panels correspond to simulations with different proportions of time-series (TS) genes; all panels include 10% batch effect genes which are sample-specific. (**E**) Absolute Kendall’s *τ* correlation coefficient between label sequences (treated as sets of integers 1,…K) and calculated pseudotimes. Error bars show 95% confidence interval over 1000 bootstraps, calculated with boot package in R. For Tempora, this calculation was performed using scipy package in python. Datasets are specified in Table 1

To compare psupertime to other classifiers, we simulated single-cell RNA-seq data to contain genes that vary over time, and also genes with other profiles (see Section 2.5). We compared psupertime’s performance against two benchmark classification methods, which also identify relevant features: multinomial regression, as a simple baseline approach to classification (Venables and Ripley, 2002) and a popular classification algorithm that performs well under many circumstances (Caruana *et al.*, 2008). Both classifiers give measures of importance for each variable (see Section 2); we used these to determine how well the classifiers identified time-series genes. We found that the coefficients identified by psupertime identify time-series genes more precisely than the benchmark classifiers (Fig. 2D, Supplementary Fig. S6). In addition, psupertime is able to recapitulate the true latent time values of the cells (Supplementary Fig. S7). The other classifiers assume no structure across the labels and identify any gene which is helpful for distinguishing one label from another; this results in them also identifying genes with sample-specific rather than time-varying variation. The model for psupertime assumes and therefore identifies genes that vary coherently over the timepoint labels.

Unsupervised projection techniques are commonly applied to analyse time-series single-cell RNA-seq data. We therefore compared the cell-level orderings identified by psupertime with those from three alternative, unsupervised pseudotime techniques: projection onto the first PCA component, as a simple, interpretable baseline; Monocle 2 (Qiu *et al.*, 2017b), which is widely used, shown to perform well in a benchmark study (Saelens *et al.*, 2019) and permits the selection of a starting point; and slingshot (Street *et al.*, 2018), which was also shown to perform well (Saelens *et al.*, 2019) and allows both the start and end point of a trajectory to be selected (it is therefore semi-supervised). Applied to the acinar cells, low-dimensional embeddings of the data (including PCA) indicate that while donor-specific factors account for much of the variation, very little transcriptional variation is related to age (Fig. 2A and B; Supplementary Fig. S8). Acinar cell orderings identified by the benchmark methods are not consistent with the known label sequence (Fig. 2C and E). In contrast, the one-dimensional projection learned by psupertime (Fig. 2C) successfully orders the cells by donor age (Kendall’s *τ* correlation coefficient 0.86, which quantifies the concordance between two orderings), while providing a sparse interpretable gene signature related to age.

In addition to the acinar cells, we compared psupertime to the three alternative methods on four further datasets, as specified in Table 1. The correlation of the orderings from the benchmark methods with the labels varies considerably depending on the dataset (Supplementary Table S2), and in particular, depending on the extent of variation unrelated to the labels (Supplementary Fig. S8): both Monocle 2 and PCA show Kendall’s *τ* values of 0.12 or below for the human germline dataset (Li *et al.*, 2017; Supplementary Fig. S9), in comparison to values of at least 0.71 for the human embryonic stem cells (ESCs) dataset (Petropoulos *et al.*, 2016; Supplementary Fig. S10). In all datasets considered, the cell ordering given by psupertime has a higher correlation with the known label sequence than the other pseudotime methods (Fig. 2E). The pseudotime methods used for comparison do not use the timepoint label as input, so it is not surprising that psupertime is better able to recapitulate the label orderings. However, considering that unsupervised methods are frequently used to analyse time series and other ordered data, this comparison is relevant for users. Where genes and processes associated with time labels are the primary interest, our analysis shows that unsupervised techniques alone are not appropriate (see also Supplementary Results S6).

Many datasets comprise samples composed of multiple distinct cell types. In a time-series experiment, this could in principle make it more difficult for psupertime to identify relevant genes: time-related signal for one cell type could be diluted when cell types are analysed together. To test this, we generated synthetic time-series data from multiple cell types, modelling genes that varied over time both globally, and individually within cell types (see Section 2.6). We found that psupertime is best able to identify globally varying genes when applied to all cell types together, and best able to identify cell-type-specific genes via application to each cell type individually (see Supplementary Results S5). In addition, we applied psupertime to a biological dataset comprising multiple distinct trajectories leading to different cell fates, specifically reprogramming mouse embryonic fibroblast cells (MEFs) to induced pluripotent stem cells (iPSCs; Schiebinger *et al.*, 2019). We identified two clear branches (Supplementary Fig. S20): one branch corresponding to reprogramming from MEFs to iPSCs, and one to reprogramming from MEFs to stromal cells. We then applied psupertime three times: to the entire dataset; to the iPSC branch; and to the stromal branch. In each case, we trained psupertime using the experimental days as labels. psupertime identified relevant genes for the global process (e.g. *Dppa5a*; Lee *et al.*, 2014), for reprogramming to iPSCs (e.g. *Cd24a*; Shakiba *et al.*, 2015) and for reprogramming to non-pluripotent cells (e.g. *Xist*; Minkovsky *et al.*, 2012; Supplementary Fig. S21). Taken together, these results show that sensible use of psupertime can identify both globally varying and cell-type-specific time-varying genes. (Details of both analyses are given in Supplementary Results S5.)

Typical workflows for single-cell RNA-seq data first restrict to highly variable genes. If the data are instead first restricted to genes that correlate strongly with the sequential labels, the relative performance of the benchmark methods might improve. Despite the selection of genes that correlate with the labels, psupertime consistently outperforms unsupervised methods in terms of identifying individual cell orderings (Supplementary Results S2). This illustrates that the genes identified by psupertime as most relevant to the process are not necessarily those with highest correlation; for example, genes with expression profiles like *AMY2B* in Figure 1E show a non-linear, step-like expression profile, which results in a correlation of 0.11 with the condition labels. Despite low correlation, such genes were nonetheless found to be useful for cell ordering and suggest that psupertime discovers meaningful non-linear structure in the data.

The time taken for psupertime to run varies over the five test datasets from 4 s for a dataset with ≈300 cells, to 32 s for one with ≈1500 cells (Table 2). We empirically observe a linear runtime dependency for the dataset size in terms of number of cells (∼5 min/10k cells). While maintaining classification accuracies of between 43% and 98%, psupertime uses a small set of genes: for example, for a classification accuracy of 76% on 10% of the acinar cells held out for testing, psupertime uses 10% of the input genes (Table 2). psupertime is based on a form of penalized linear regression. We show that the ordinal logistic model, rather than a linear model based on regarding the sequential labels as integers, is both the natural and the best-performing model for this problem (see Supplementary Results S2).

**Table 2:** psupertime performance and timings on comparison datasets

Dataset name	Accuracy (%)	Time taken (s)	Sparsity (%)
Acinar cells	75.7 ± 1.1	5.5 ± 0.41	90.6 ± 1.8
Human germline, F	43.4 ± 1.5	25 ± 0.73	80.4 ± 4.9
Embryonic beta cells	78.5 ± 0.9	19 ± 0.67	96.4 ± 0.4
Human ESCs	97.6 ± 0.2	35 ± 0.80	90.0 ± 1.1
MEF to neurons	89.6 ± 1.7	4.7 ± 0.062	96.6 ± 0.4

*Note*: Mean and standard deviation of psupertime accuracy, timing and sparsity calculated over 10 random seeds.

## 4 Discussion

The number of studies using single-cell RNA-seq is increasing exponentially (Saelens *et al.*, 2019), and many of these include time-series labels. psupertime is explicitly designed to take advantage of such a setting, complementing unsupervised pseudotime techniques. The presence of time-series labels allows a simple, regression-based model to identify relevant cell orderings; here, the more sophisticated pseudotime approaches required for unlabelled data identify the principal variation in the data, rather than that associated with the labels. The potential asynchrony of dynamic processes is expected to affect classification performance. Specifically, we expect the misclassification rate to increase with stronger asynchrony. While poor classification performance can have other causes than asynchrony, we recommend to consider asynchrony as a possible cause for poor psupertime classification performance and to resort to other dedicated tools/experiments to investigate possible asynchrony. psupertime uses L1 regularization to obtain a small set of reported genes. However, this may result in exclusion of other relevant genes: where there are multiple highly correlated genes that are predictive of the sequential labels, L1 regularization will tend to result in only one of these genes being reported, and produce zero coefficients for other correlated genes. This issue can be addressed by calculating the psupertime ordering, and reviewing all genes that have high correlations with the genes identified by psupertime. Alternatively, a simple extension to psupertime would allow training with a combination of L1 and L2 penalties (the elastic net), resulting in a compromise between sparsity and prediction performance. psupertime could possibly benefit from alternative normalization techniques, such as regularized negative binomial regression resulting in Pearson residuals (Butler *et al.*, 2018), as well as combination with RNA velocity-based pseudotime (Bergen *et al.*, 2020). psupertime is applicable to any experimental design with sequential labels, most obviously time series but also to biological questions regarding drug dose–response, and disease progression. psupertime could further be used in situations without experimental labels by combining with unsupervised techniques (see Supplementary Results S3) or to align new data to orderings learned from alternative processes or separate lineage branches (see Supplementary Results S5 and Figs. S19–S22). More broadly, we have used it to improve dimensionality reduction (see Supplementary Results S4) and are developing extensions including to additional single-cell technologies such as mass cytometry (see Supplementary Results S6). This demonstrates the potential of ordinal regression models for further methodological developments. psupertime has wide applicability and will enable quick and effective identification of the genes and profiles relevant to state sequences of biological processes in single-cell RNA-sequencing data. We have developed an R package available for download at github.com/wmacnair/psupertime.

*Financial Support*: none declared.

*Conflict of Interest*: none declared.

## Supplementary Material

btac227_Supplementary_Data
