# Supplementary material for: psupertime: supervised pseudotime analysis for time-series single-cell RNA-seq data
Source: Bioinformatics. 2022 Jun 27;38(Suppl 1):i290–8. doi: 10.1093/bioinformatics/btac227 (PMC9235474; doi:10.1093/bioinformatics/btac227)
Supplement: btac227_Supplementary_Data [file btac227_supplementary_data.zip › btac227-Suppl_data/Macnair.56.supp.pdf]

.

5 Supplementary Tables

Supp Table 1. Correlations of pseudotimes with known labels, using genes correlated with labels as input.
See subsection 2.8 for details of calculations.

Dataset	Method	Spearman's ρ	Kendall's τ
Acinar cells	PCA	0.68	0.54
Acinar cells	Monocle2	0.73	0.58
Acinar cells	slingshot	0.61	0.53
Acinar cells	Tempora	0.88	0.85
Acinar cells	psupertime	0.94	0.82
Embryonic beta cells	PCA	0.90	0.76
Embryonic beta cells	Monocle2	0.92	0.79
Embryonic beta cells	slingshot	0.93	0.82
Embryonic beta cells	Tempora	0.60	0.49
Embryonic beta cells	psupertime	0.98	0.90
Human ESCs	PCA	0.81	0.67
Human ESCs	Monocle2	0.89	0.77
Human ESCs	slingshot	0.48	0.40
Human ESCs	Tempora	0.89	0.80
Human ESCs	psupertime	0.97	0.87
Human germline, F	PCA	0.51	0.39
Human germline, F	Monocle2	0.49	0.36
Human germline, F	slingshot	0.49	0.40
Human germline, F	Tempora	0.73	0.59
Human germline, F	psupertime	0.87	0.72
MEF to neurons	PCA	0.86	0.71
MEF to neurons	Monocle2	0.89	0.73
MEF to neurons	slingshot	0.74	0.62
MEF to neurons	Tempora	0.99	0.98
MEF to neurons	psupertime	0.97	0.88

Supp Table 2. Correlations of pseudotimes with known labels, using highly variable genes as input
See subsection 2.8 for details of calculations.

Dataset	Method	Spearman's ρ	Kendall's τ
Acinar cells	PCA	0.56	0.40
Acinar cells	Monocle2	0.57	0.43
Acinar cells	slingshot	0.66	0.57
Acinar cells	Tempora	0.87	0.85
Acinar cells	psupertime	0.96	0.86
Embryonic beta cells	PCA	0.88	0.73
Embryonic beta cells	Monocle2	0.93	0.80
Embryonic beta cells	slingshot	0.93	0.83
Embryonic beta cells	Tempora	0.59	0.46
Embryonic beta cells	psupertime	0.98	0.90
Human ESCs	PCA	0.84	0.71
Human ESCs	Monocle2	0.87	0.75
Human ESCs	slingshot	0.89	0.78
Human ESCs	Tempora	0.90	0.82
Human ESCs	psupertime	0.97	0.87
Human germline, F	PCA	0.10	0.08
Human germline, F	Monocle2	0.17	0.11
Human germline, F	slingshot	0.34	0.26
Human germline, F	Tempora	0.70	0.55
Human germline, F	psupertime	0.91	0.78
MEF to neurons	PCA	0.87	0.72
MEF to neurons	Monocle2	0.87	0.70
MEF to neurons	slingshot	0.74	0.61
MEF to neurons	Tempora	0.96	0.91
MEF to neurons	psupertime	0.97	0.88

6 Supplementary Figures

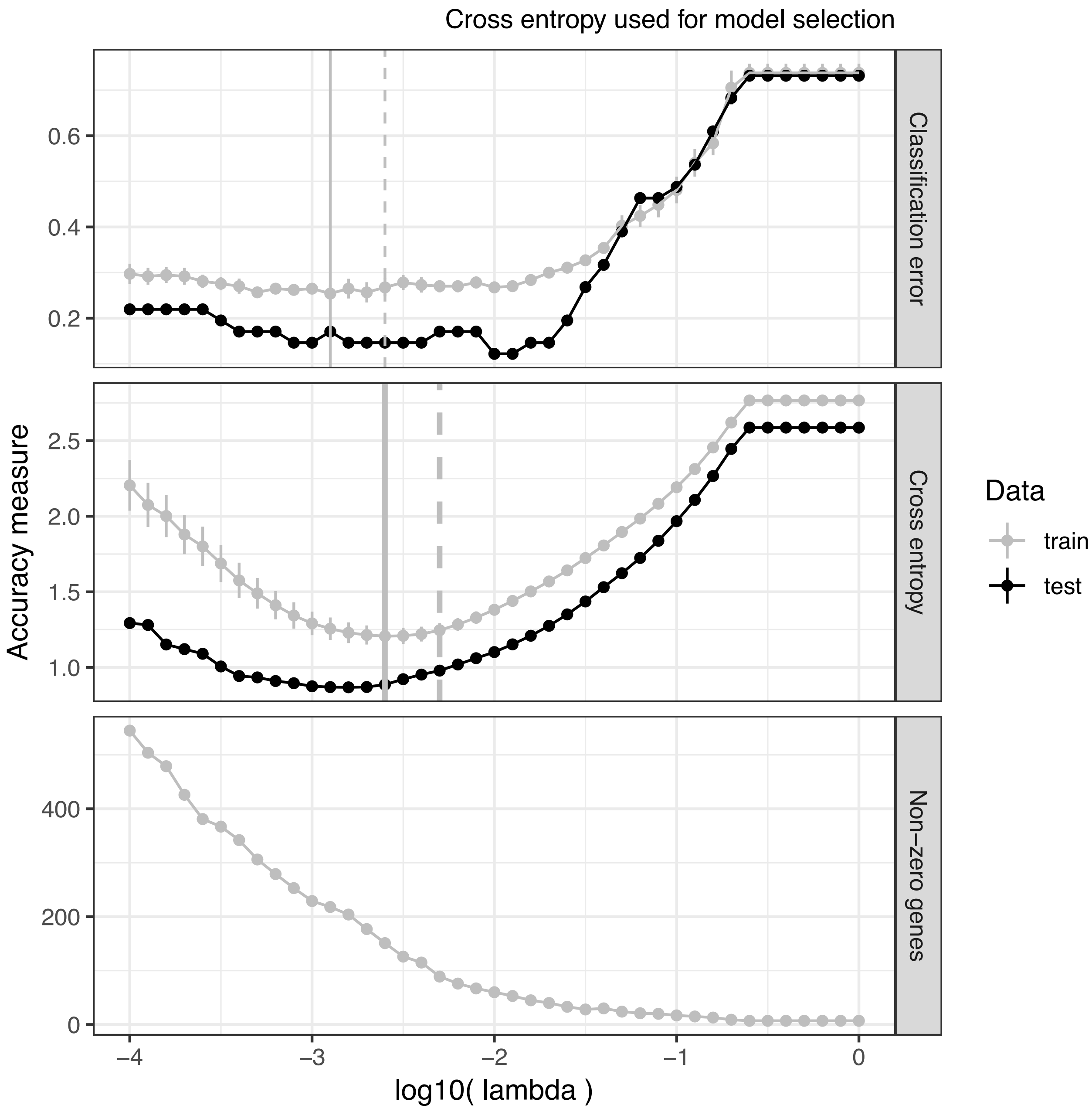

Supp Fig 1. Training and test performance of **psupertime** applied to acinar cells. x-axis is $\log$ value of λ, the weight given to the L_1 penalty. y-axis is a measure of performance. Classification error is the proportion of cells for which `psupertime` predicted the incorrect label. Cross entropy is a measure of how confidently `psupertime` predicted the correct label, and has low values when a correct label is predicted with high probability. Non-zero genes is the number of genes with non-zero coefficients in this model. The grey trend line shows the mean performance measure over the 5 folds in the training data, with vertical whiskers showing the s.e. of the mean. The black trend line shows performance on 10% of data not used for training. Vertical grey lines show the value of lambda with the best performance on the training data (solid) and within one s.e. of the best performance (dashed line); here, the lambda corresponding to the dashed line was selected. The measure used for selection of λ (cross-entropy) is indicated with thicker vertical grey lines.

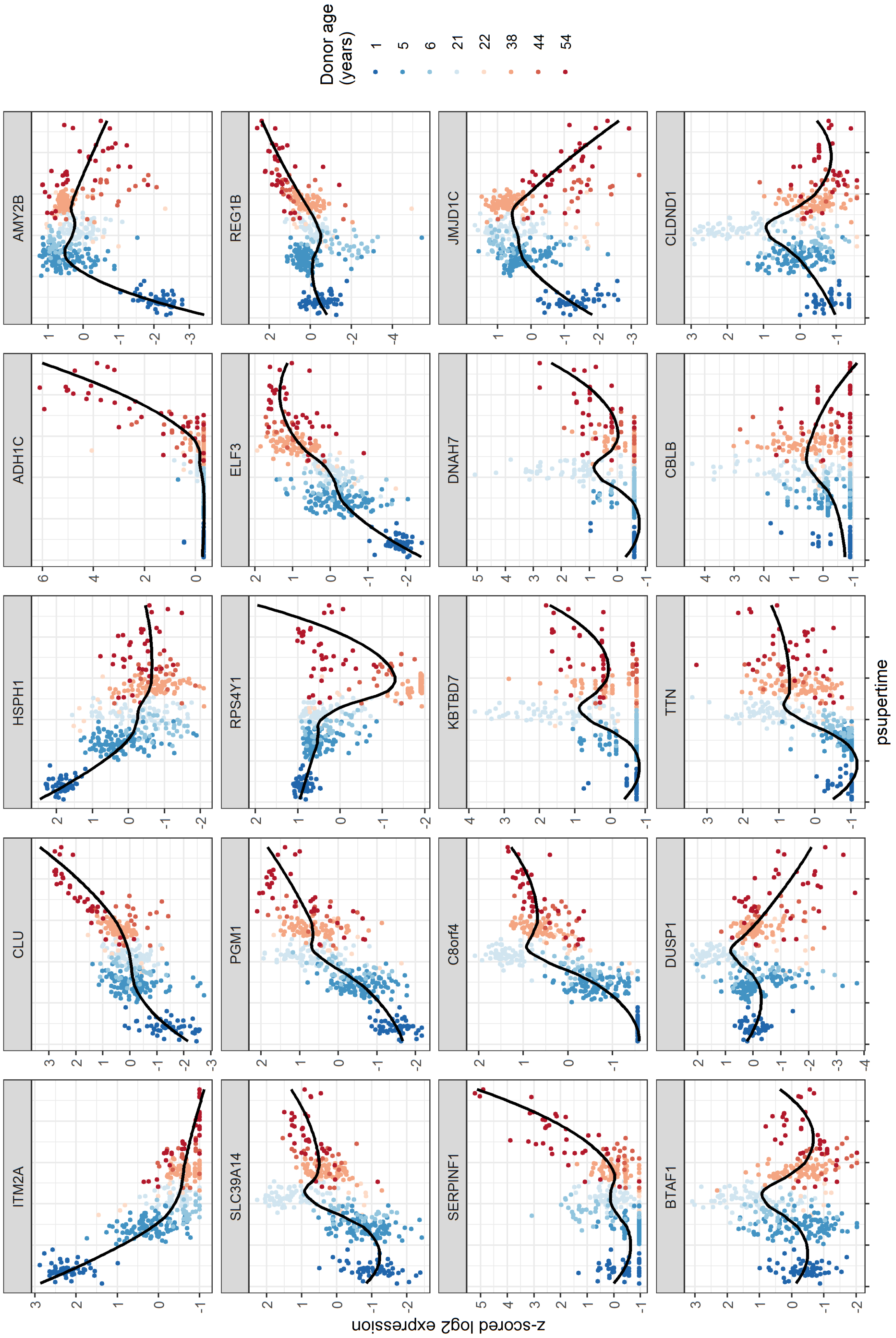

Supp Fig 2. Profiles of top genes identified in acinar cells by **`psupertime`**. Results of `psupertime` applied to 411 acinar cells. 20 genes with highest absolute coefficients, plotted against `psupertime` pseudotime. x-axis is the values from projections of each cell by `psupertime`. y-axis is smoothed, z-scored log pseudocounts for each cell. Colours indicate ordered labels. Black line is smoothed curve as fit by `geom_smooth` in the R package `ggplot2` .

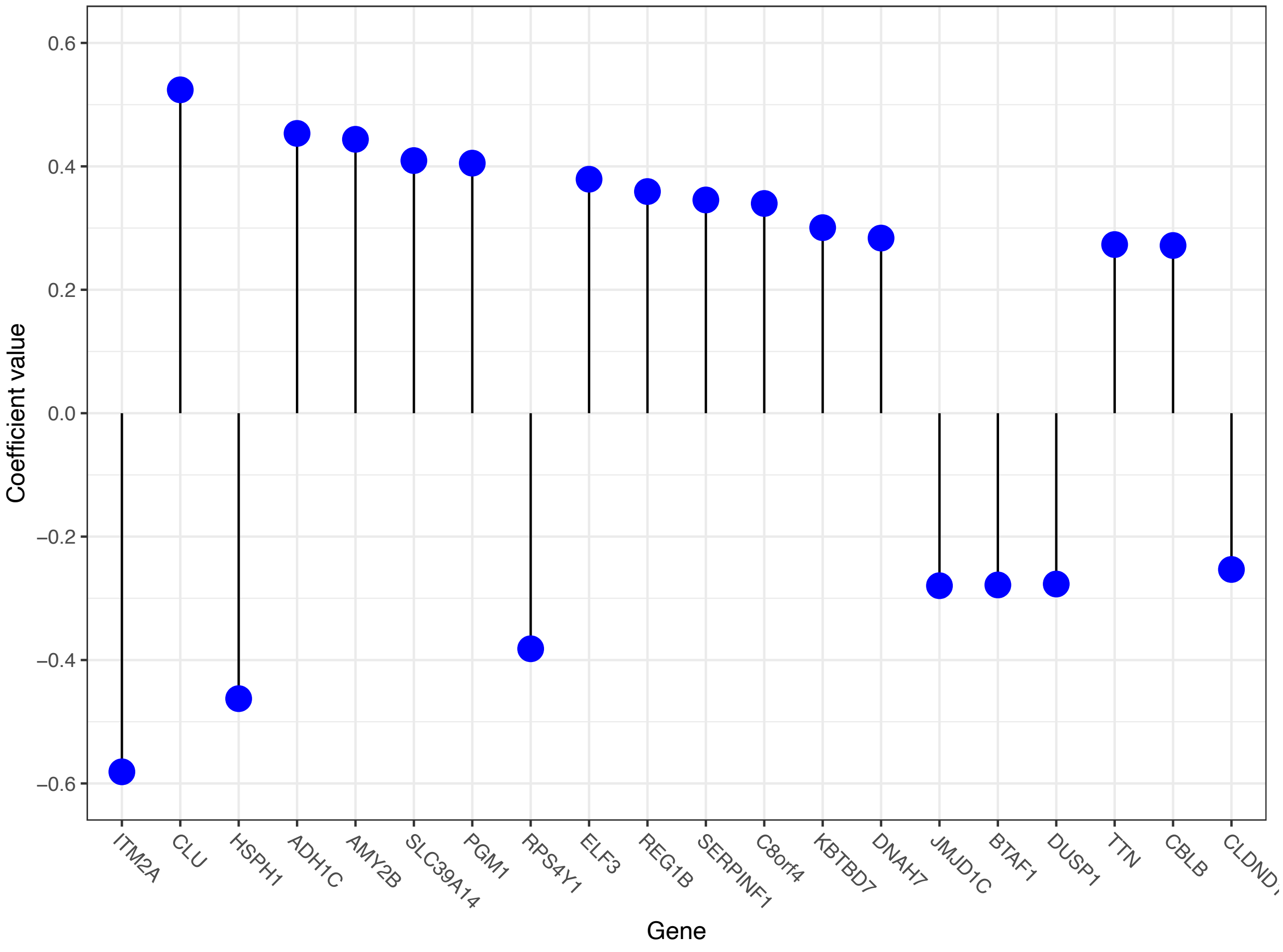

Supp Fig 3. Top genes identified in acinar cells by **`psupertime`**. 20 genes with largest absolute ordering coefficients β_i, subject to $\beta_i > 0.05$, ordered by absolute value. Non-zero coefficients correspond to genes relevant to the process underlying the condition labels, and coefficient indicates strength and direction of the effect of this gene on the predicted label.

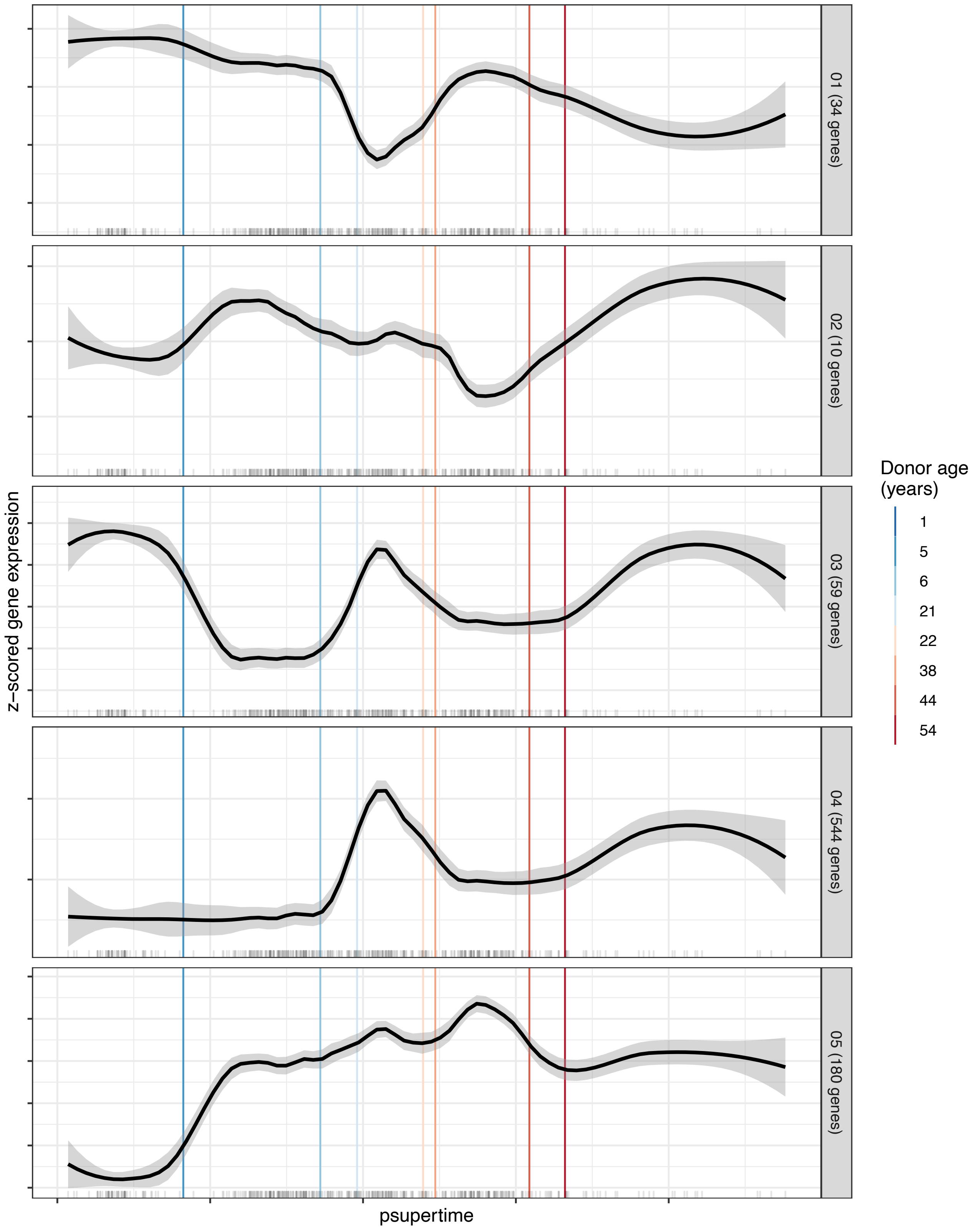

Supp Fig 4. Mean expression profiles of gene clusters, ordered by **`psupertime`**. Complete linkage hierarchical clustering was applied to 827 highly variable genes, with $k = 5$. Black line is smoothed curve as fit by `geom_smooth` in the R package `ggplot2`. Clusters ordered by correlation between mean expression over the gene cluster, and the pseudotime values learned by `psupertime`. Vertical lines indicating predicted cutoffs separating labels. Marks on x-axis denote pseudotime values of individual cells.

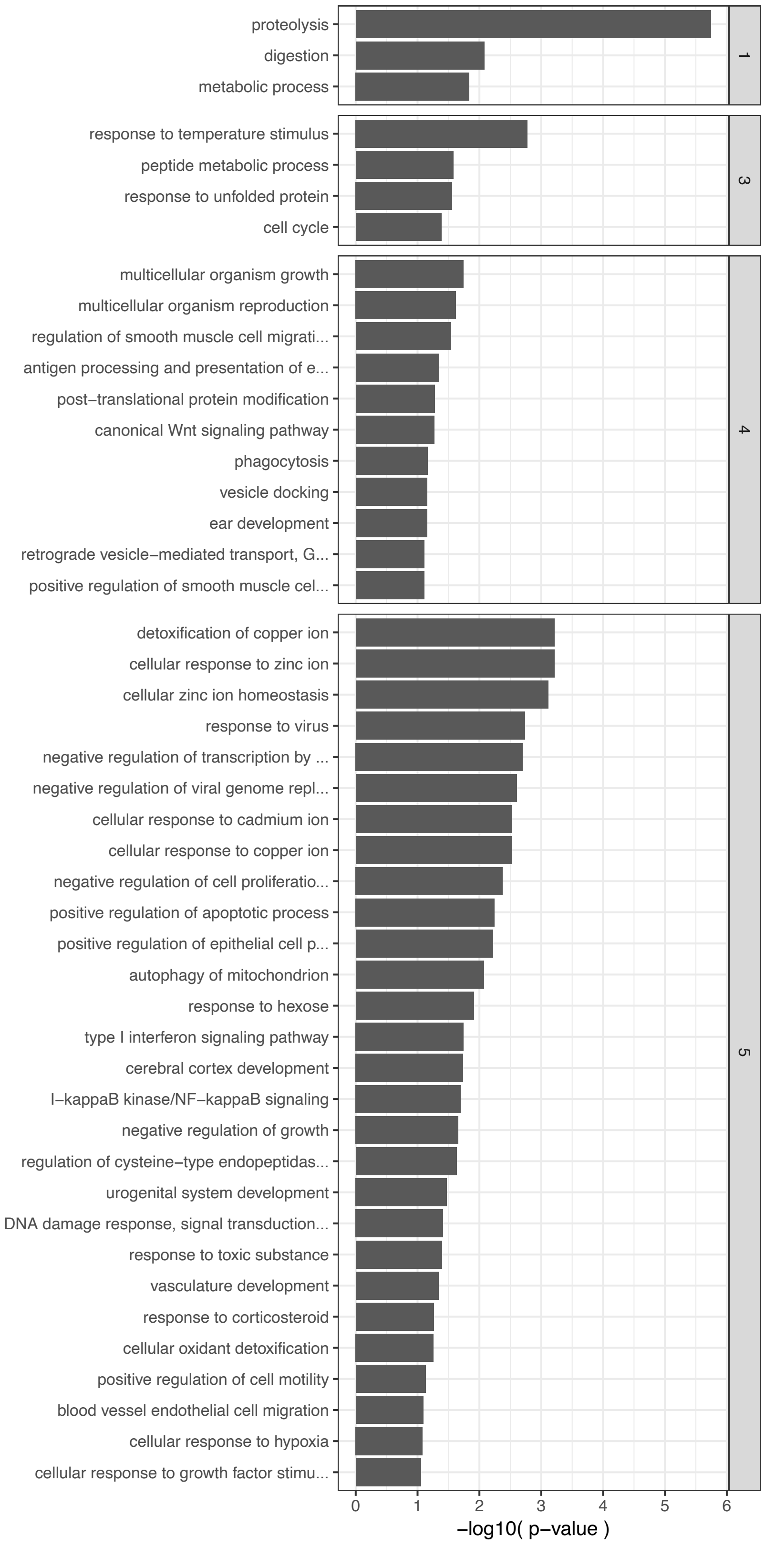

Supp Fig 5. Enriched GO terms associated with gene clusters, ordered by **`psupertime`**. Biological process GO terms enriched in each gene cluster, relative to all other gene clusters. x-axis is uncorrected p-values for Fisher's exact test. Only GO terms with at least 5 genes annotated in the cluster and p-value < 0.1 are shown (this results in no GO terms shown for cluster 2). See subsection 2.9 for details of calculation.

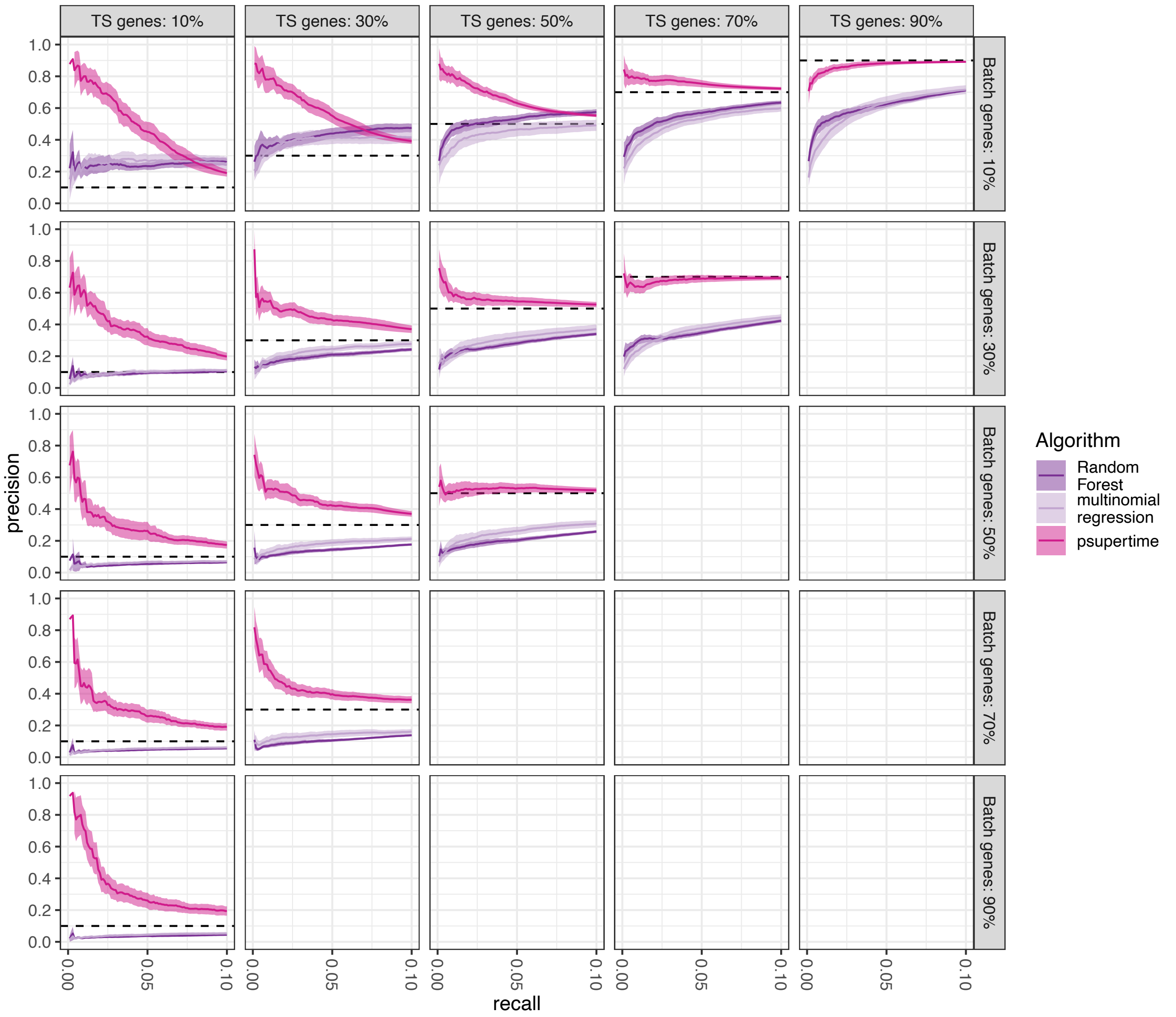

Supp Fig 6. Performance of `psupertime` and benchmark classifiers in identifying simulated time-series genes. Precision-recall curves based on identification of time-series genes via variable importance measures for each method (see subsection 2.7). Line and area show mean and ± 2 standard error respectively, over 20 simulations. Recall is limited to range 0% to 10%, to test identification of small, relevant subset. Columns correspond to simulations with different proportions of time-series (TS) genes; rows correspond to simulations with different proportions of "batch-effect genes" which are sample-specific. Black dashed line shows proportion of genes which are true, i.e. rate of success which would be achieved by random guessing.

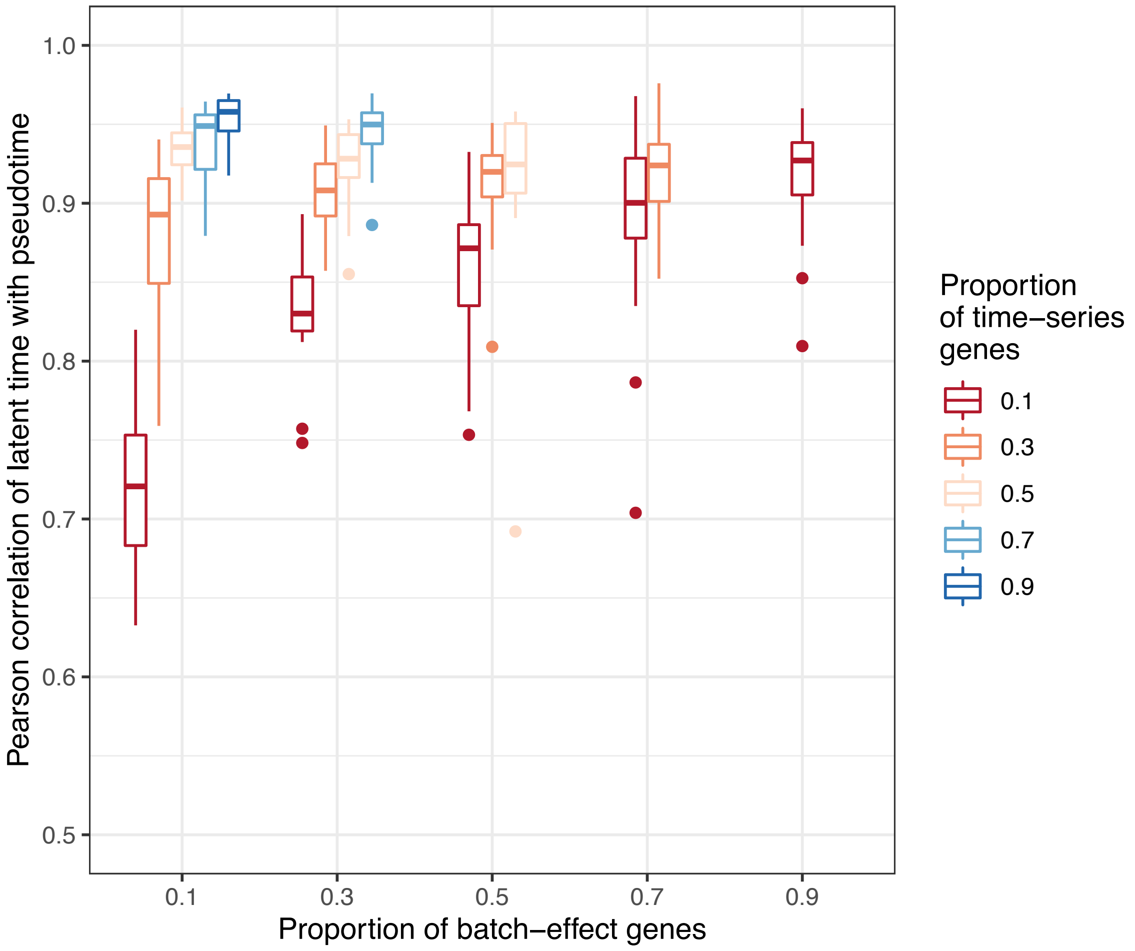

Supp Fig 7. Correlation of pseudotimes learned by `psupertime` and true simulated latent time values. Boxplots showing distribution of Pearson correlations between pseudotimes and true latent times for each cell, over 20 simulations (see subsection 2.5). Centre line corresponds to median, lower and upper hinges correspond to 1st and 3rd quartiles, whiskers extend to smallest/largest values no further than $1.5 \times IQR$ from hinge. Colours correspond to simulations with different proportions of time-series (TS) genes; x-axis corresponds to simulations with different proportions of "batch-effect genes" which are sample-specific. Performance with low proportion of both time-series and batch-effect genes is lowest, as here there is the least time-related signal in the data.

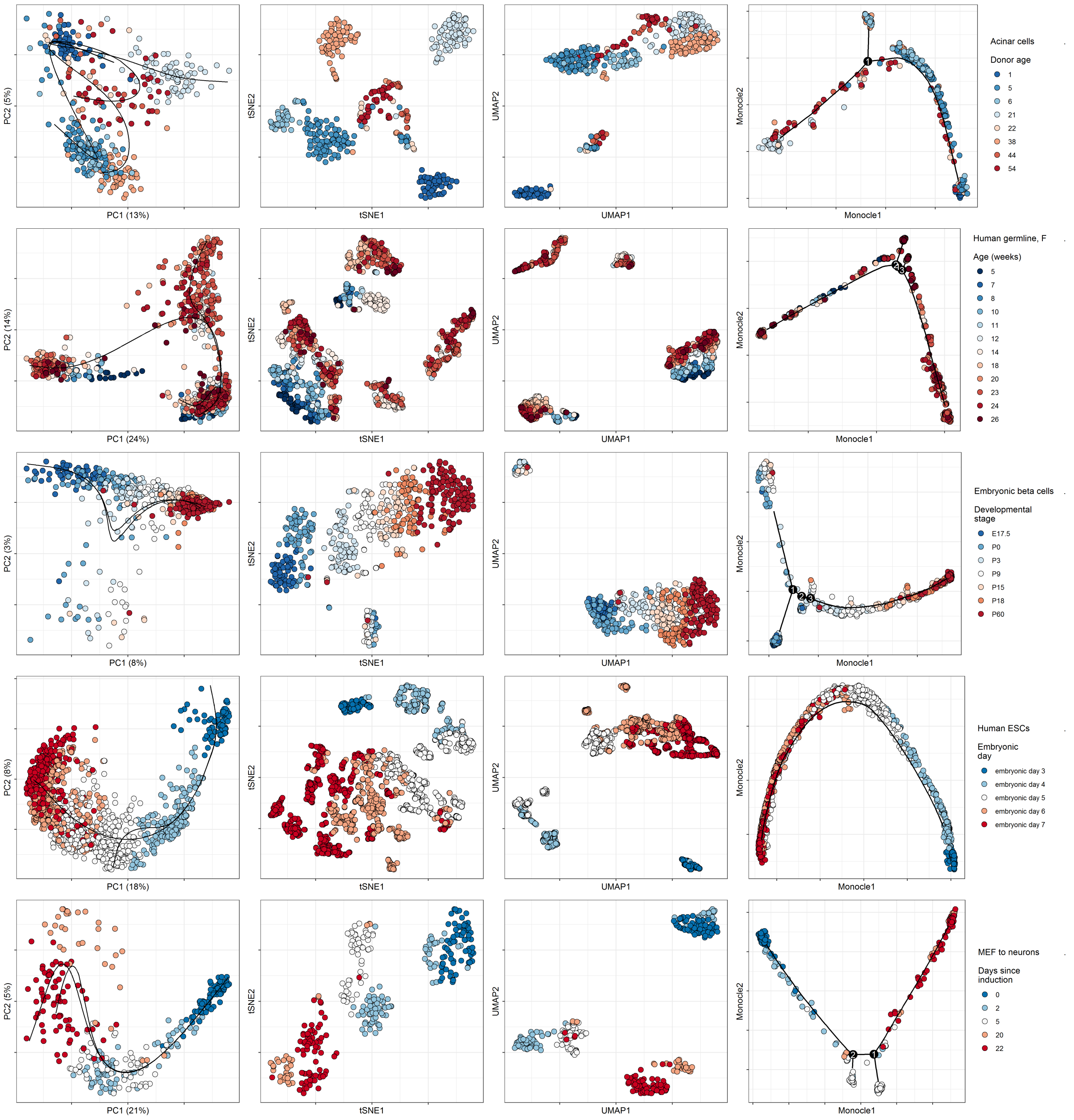

Supp Fig 8. Dimensionality reduction methods applied to comparison datasets. Rows correspond to datasets detailed in Table 1. Columns correspond to dimensionality reduction methods, with annotations by comparator pseudotime inference techniques. First column corresponds to projection into the first two principal components, annotated with curves learned by `slingshot` (Street et al., 2018), which are used as pseudotime. Second column shows projection by t-SNE, using default parameters . Third column shows projection by `UMAP`, using default parameters . Fourth column shows dimensionality reduction by `Monocle 2`, annotated with the tree it learns and which is used for pseudotime inference (Qiu et al., 2017b).

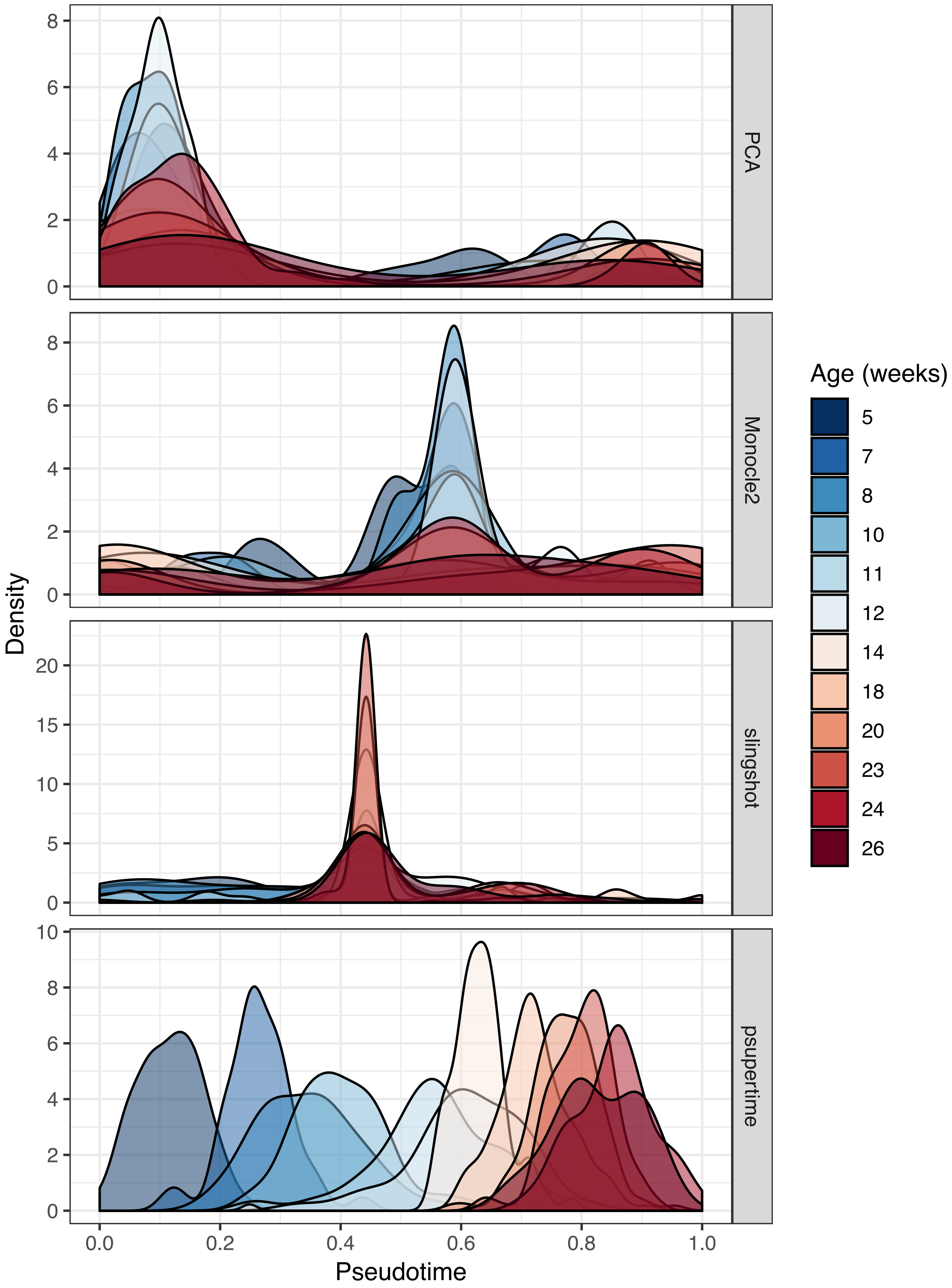

Supp Fig 9. Benchmark methods applied to female human germline data. Female human germline data; colours indicate weeks post-fertilization. x-axes are the pseudotimes generated by each method, scaled to take values between 0 and 1. y-axes are density of the distributions for each label used as input, as calculated by the function `geom_density` in the `R` package `ggplot2`.

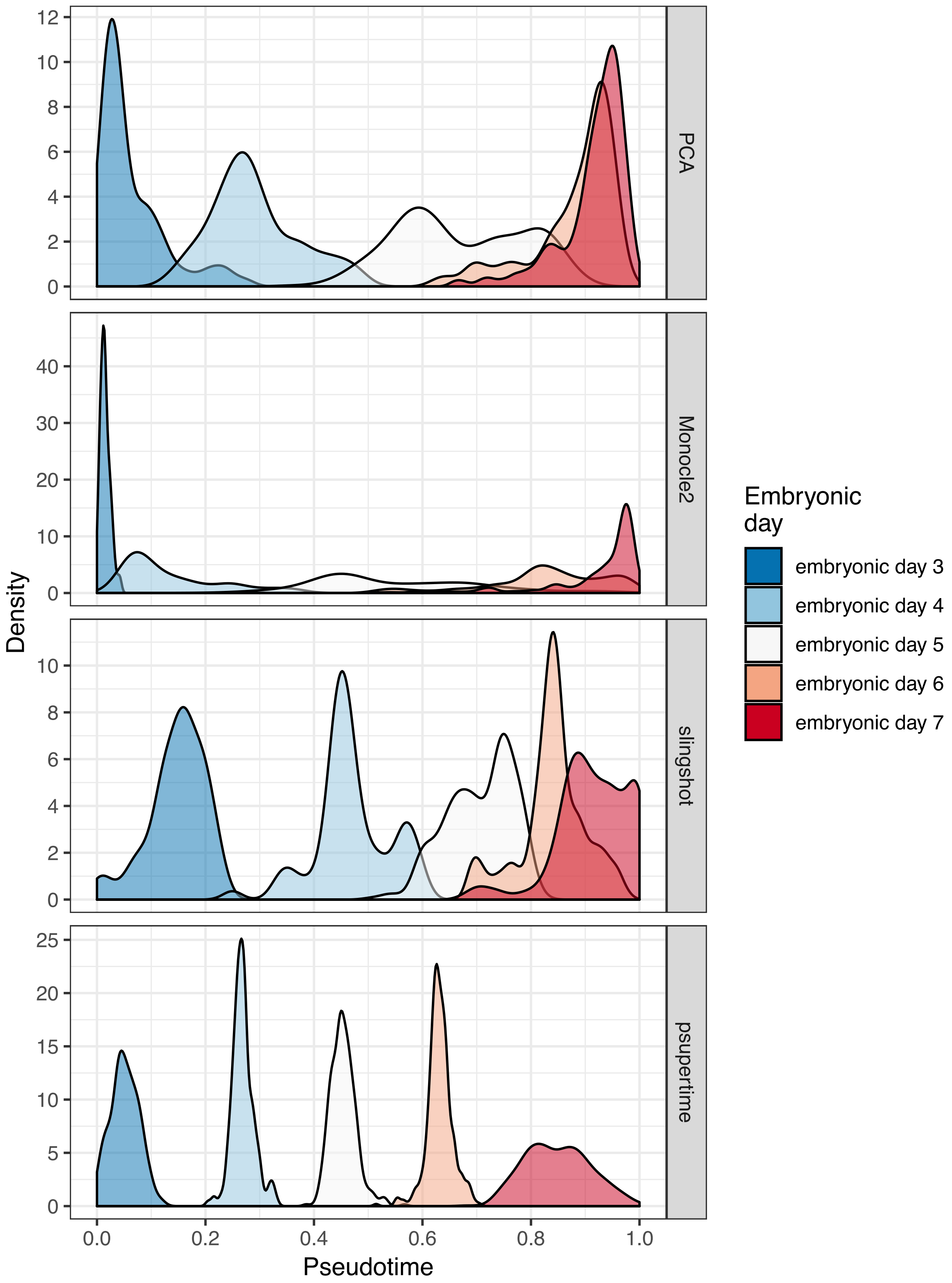

Supp Fig 10. Benchmark methods applied to human ESC data. Human embryonic stem cell (ESC) data; colours indicate embryonic days. x-axis is the pseudotime generated by each method, scaled to take values between 0 and 1. y-axes are density of the distributions for each label used as input, as calculated by the function `geom_density` in the `R` package `ggplot2` (Wickham, 2016).

7 Supplementary Results

Supplementary Results 1: **psupertime** is robust to label perturbations

The set of condition labels used to train psupertime is critical to both the geneset learned by psupertime, and the accuracy which can be achieved. If the condition labels progress at a different rate to the underlying biological process, the flexibility in defining thresholds between labels means that psupertime's performance should not be affected. However, if some of the labels are mislabelled, this may be reflected in reduced classification accuracy for a subset of the labels.

We demonstrate that the performance of psupertime is robust to a number of perturbations which are plausibly relevant to analysis. We measured the test accuracy of psupertime performance under the following perturbations:

1. using different random seeds for the cross-validation folds;
2. selecting at random a pair of neighbouring labels, and swapping their order;
3. completely randomizing the label order; and
4. randomizing the labels of all cells.

We analysed psupertime's performance on the datasets detailed in Table 1. For each dataset, we first restricted to highly variable genes, using the default settings for psupertime. For each of the perturbations, we ran psupertime twenty times and recorded relevant performance measures: test classification error, test cross-entropy, and sparsity.

The performance of psupertime is robust to choice of cross-validation fold, resulting in only small variations in test classification error (Supp Fig 11). (We note that classification error is a volatile measurement, as it is based on 10% of a relatively small number of cells.) Swapping pairs of neighbouring labels results in a small increase in psupertime classification error, indicating that small experimental flaws or perturbations do not result in substantial reduction in performance for other labels.

Where the order of labels is randomized, the performance of psupertime is reduced, but for some datasets this reduction was small. This may indicate that within the large number of highly variable genes used as input (between ≈ 800 and ≈ 2900; see Table 1), there are sufficient genes to recapitulate a given order of a relatively small number of labels, for a relatively small number of observations.

The number of non-zero genes required to achieve a given level of classification error (i.e. the sparsity) is more variable. Where the cell labels are completely randomized, psupertime consistently identifies no genes as being relevant to the ordering, showing that it does not find spurious genes where there is no structure to the data.

The perturbations discussed here correspond to potential mislabelling of the data, which could pose a challenge to psupertime. A further challenge comes from data containing branching structure, in which progress along the biological process is accompanied by a bimodal (or multimodal) distribution of expression for some genes. This results in increased variance in gene expression, which could make pseudotime inference more difficult. Here, we advise applying psupertime both globally and to branches (or equivalently celltypes) identified by complementary methods (see discussion in Supp Results 5).

psupertime could be affected by the presence of cell sub-populations unrelated to the sequential labels. If this population is consistent across the labels, we expect that psupertime would identify relevant genes, although it would have lower accuracy due to being unable to accurately place the unrelated cells. Where there are variable unrelated subpopulations, filtering them out before applying psupertime should improve performance. Variability in cell population which is *related* to the sequential labels is not expected to affect the performance of psupertime.

Taken together, these results indicate that the performance of psupertime is robust, in particular to perturbations in the labels used.

Supplementary Results 2: **psupertime** identifies better cell orderings irrespective of gene selection method

Single cell RNA-seq datasets include measurements of thousands of genes, or features, many of whose measurements may be noisy, or irrelevant to the processes generating the dataset. To identify relevant features, a subset of genes is selected according to statistical criteria. The default criteria implemented in psupertime are those proposed by Lun *et al.* and implemented in the R package scran: they note a consistent relationship between the mean and variance of log gene expression data, and select genes which show above average variance given their mean expression. Such an approach is useful to identify relevant genes in an unbiased way, however it does not take into account the information given by sequential labels. Selecting genes which co-vary with the sequential labels should restrict the data to a subset relevant to the labelled process, and not just the genes varying over the dataset (which might therefore include genes affected by batch effects, for example). Co-varying genes can be selected by calculating correlation values, or more generally by identifying genes where a significant proportion of expression variance is explained by the labels (e.g. via ANOVA).

By restricting to genes co-varying with the process, selecting such genes could in principle affect the results of comparisons between psupertime and unsupervised methods. We considered four approaches for selecting relevant genes:

1. highly variable genes, following Lun *et al.* (Lun *et al.*, 2016) and using selection criteria FDR < 0.10, biological component > 0.5;
2. treating the sequential labels as integers $1, \ldots, K$, and selecting genes with absolute Spearman correlation > 0.2;
3. treating the sequential labels as integers $1, \ldots, K$, and selecting genes with absolute Kendall's $\tau > 0.2$; and
4. performing ANOVA on all genes, using the labels as the group variable, and selecting genes with $p <$ 1e-20, and standard deviation > 2.

For each dataset, we identified genes on the basis of these criteria, and used these as input into psupertime and the comparator methods. The Kendall's τ correlation was calculated between the identified pseudotimes and the sequential labels, treated as integers (see subsection 2.8 for details).

The relative performances of psupertime and the benchmark methods remain broadly the same across the different methods of gene selection. In particular, under all methods of gene selection, and across all datasets, psupertime attains higher correlations than the unsupervised methods (Supp Fig 12). This indicates that even after selecting genes which co-vary with the sequential labels denoting the process of interest, it is necessary to use these labels directly in the inference procedure to obtain pseudotimes which recapitulate the label ordering.

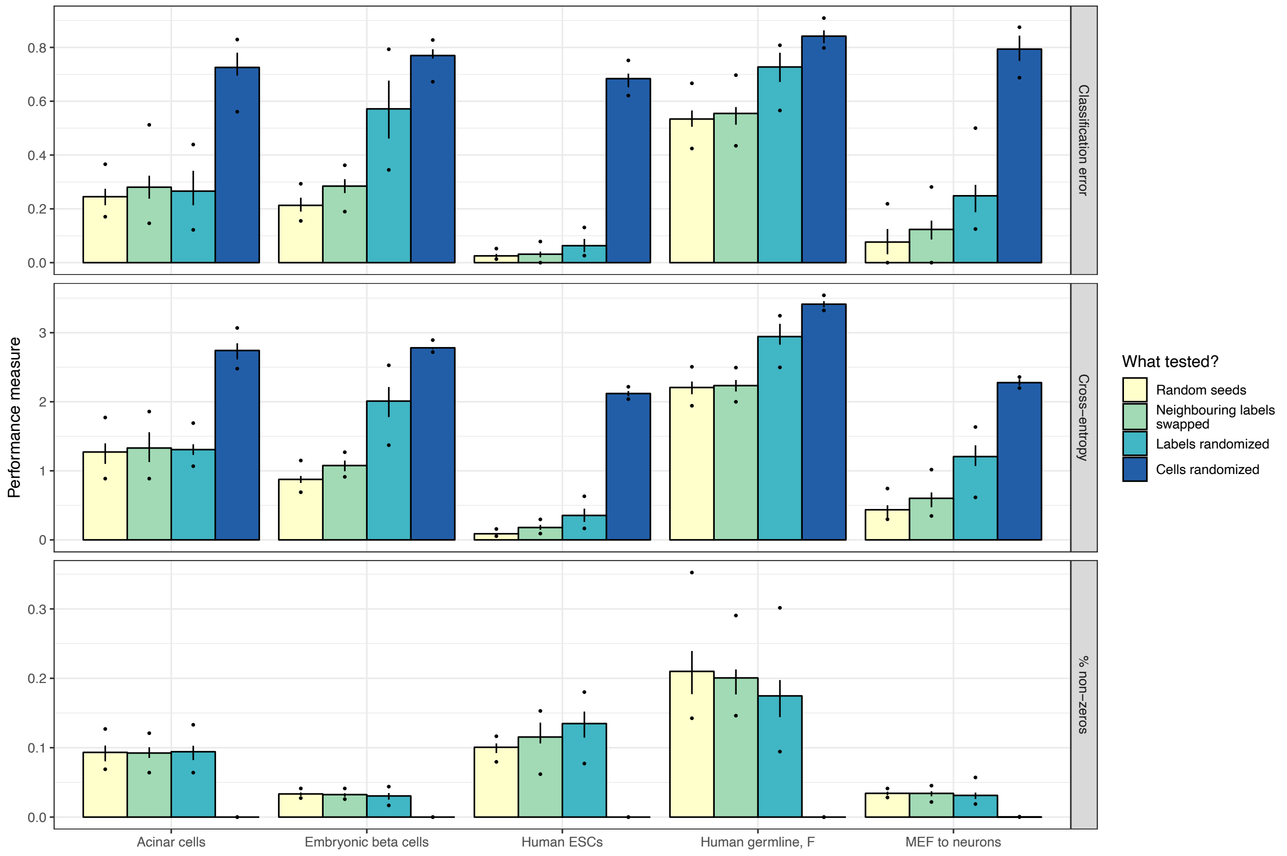

Supp Fig 11. Robustness of **`psupertime`** to perturbations of labels. Rows correspond to measures of `psupertime`'s performance: classification error is the proportion of labels correctly assigned by `psupertime`; cross-entropy is a measure of confidence in predictions, which is high when a correct label is predicted with high probability; proportion of non-zero genes indicates what proportion of the input genes for which `psupertime` identified non-zero coefficients. Both classification error and cross-entropy are shown for test data, i.e. cells which were not used to train `psupertime`. The line range shows interquartile range, dots show minimum and maximum values observed, both over 20 random runs. The x-axis corresponds to the datasets detailed in Table 1.

The need for supervised methods is reinforced by the results for LASSO regression (Tibshirani, 1996). To perform LASSO regression, we converted the sequential labels into integer values $1, \ldots, K$, and did penalized linear regression. LASSO regression and `psupertime` show similar performance in terms of ability to recapitulate the ordering of the sequential labels, as measured by Kendall's τ (Supp Fig 12), however `psupertime` is better able to classify the cells than LASSO (Supp Fig 13). These results could be expected: treating the sequential labels as integers to be regressed against, as in LASSO, is optimizing for correlation rather than separation, while the thresholds between labels give `psupertime` additional flexibility as a classifier. Taken together, these results suggest that `psupertime` is the appropriate statistical model in terms of both ordering the labels according to the sequence, and accurately labelling the cells.

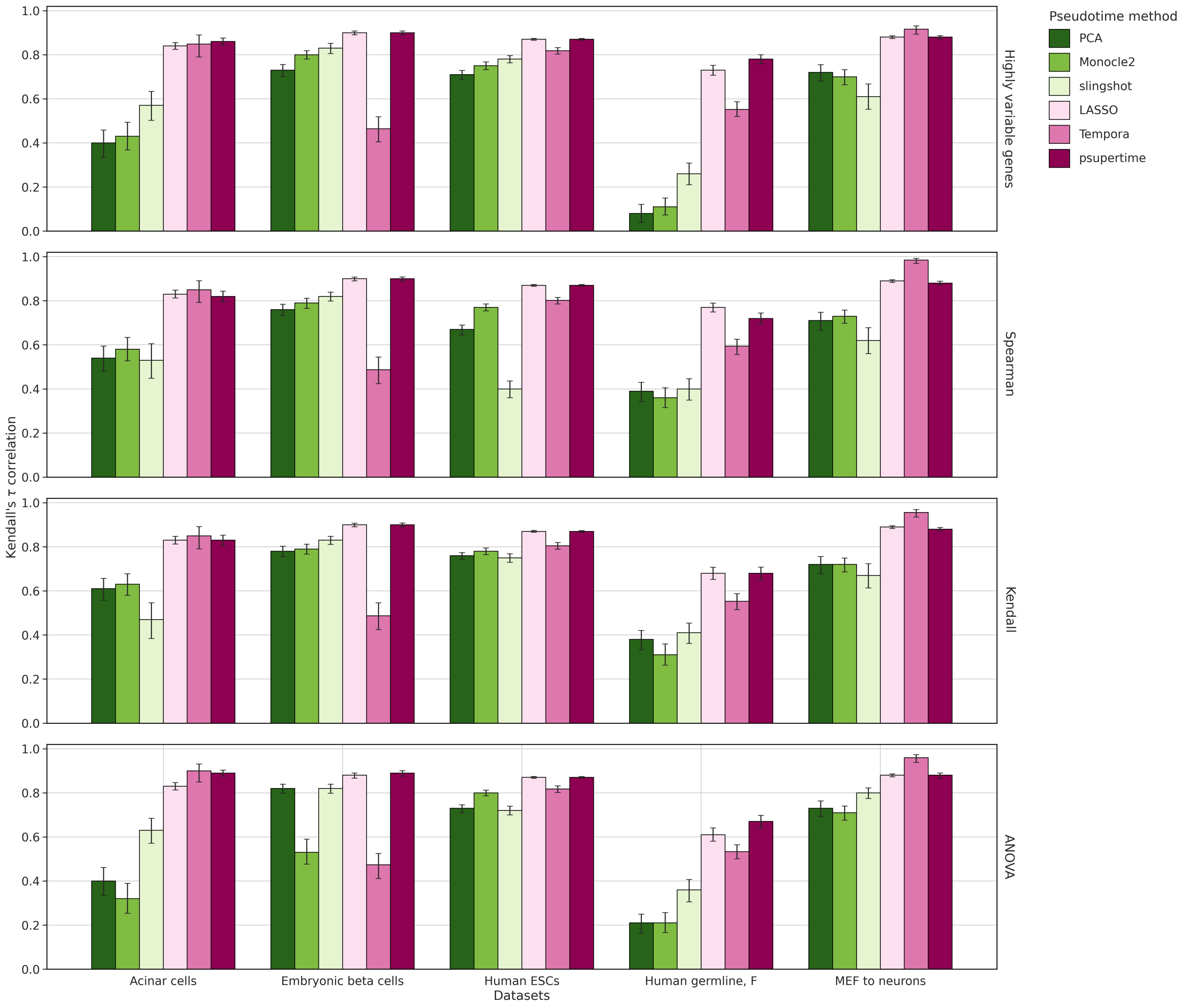

Supp Fig 12. Performance of benchmark methods under different methods for gene selection. Rows correspond to different methods for selecting genes for input into the methods. The x-axis corresponds to datasets, detailed in Table 1. Colours indicate the benchmark pseudotime inference approaches described in subsection 2.8. The y-axis shows Kendall's τ statistic, which assesses the extent of discordance between two orderings. For each combination of dataset and gene selection method, the five tested pseudotime approaches use exactly the same genes as inputs. Error bars show 95% confidence interval over 1000 bootstraps, calculated with `boot` package in `R`. For Tempora, this calculation was performed using `scipy` package in `python` (Canty and Ripley, 2017).

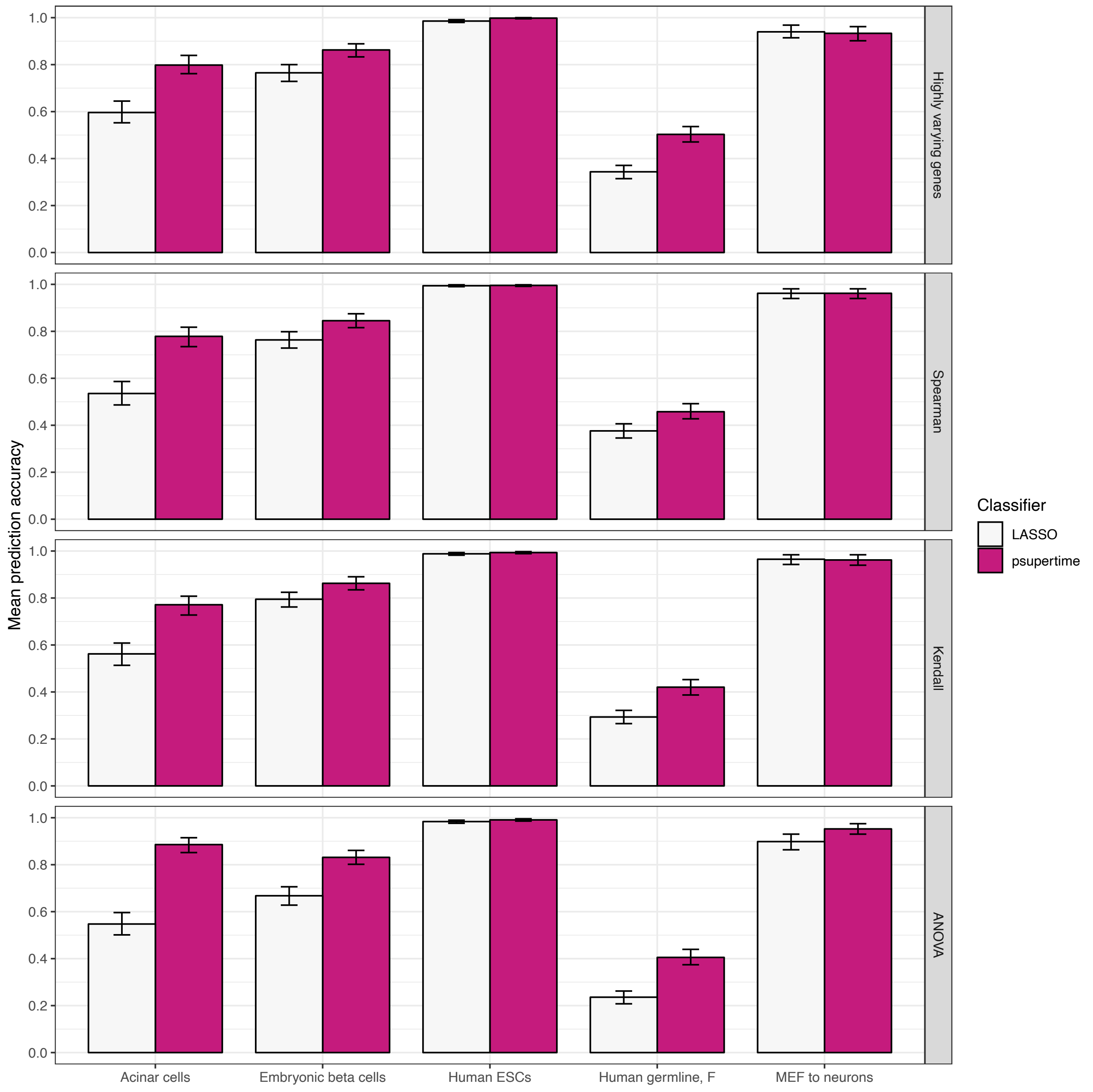

Supp Fig 13. Classification performance of LASSO and **psupertime** under different methods for gene selection. Rows correspond to different methods for selecting genes for input into the methods. The x-axis corresponds to datasets, detailed in Table 1. y-axis shows the mean prediction accuracy over all cells. Classification under LASSO is done by fitting the model, calculating the estimated value $\hat{y}$ for a given cell, and reporting the closest integer to $\hat{y}$ in $1, \ldots, K$. For each combination of dataset and gene selection method, the two tested classifiers use exactly the same genes as inputs. Error bars show 95% confidence interval over 1000 bootstraps, calculated with `boot` package in `R` (Canty and Ripley, 2017).

Comparisons of `psupertime` with unsupervised methods are not strictly fair, as the two types of approach are designed for different tasks: `psupertime` is trained to identify genes co-varying with the labels, while the other methods are not. However, unsupervised methods were previously the only methods available for investigating datasets with sequential labels, and have been used for this task; our comparisons are therefore relevant. It is precisely this use of the labels which is the major contribution of `psupertime`.

Supplementary Results 3: **`psupertime`** as a tool for exploratory data analysis of unlabelled single cell RNA-seq data

In studies without sequential condition labels, dimensionality reduction may suggest trajectories within the dataset that are of biological interest. To explore such datasets, researchers can specify a sequence of subpopulations, and use `psupertime` to identify the genes which are regulated along it.

We demonstrate this application on single cell RNA-seq data from the colon, where goblet and colonocyte cells are known to be renewed by stem cells. Supp Fig 14A shows a two-dimensional embedding of 1894 unlabelled cells from the colon (Herring *et al.*, 2017), indicating several possible trajectories

of interest. Unsupervised clustering (Supp Fig 14B) allows trajectories to be specified by the user, two of which are shown in Supp Fig 14C. We used `psupertime`, combined with clustering of genes and geneset enrichment analysis, to identify biological processes characteristic of these trajectories (Supp Fig 14D, Supp Fig 15; see subsection 2.9). Comparison of these results with the discussion in the source manuscript for the data ((Herring *et al.*, 2017)) suggests that the upper trajectory corresponds to differentiation from stem cells into colonocytes, cells responsible for absorption in the intestine, and that the lower trajectory corresponds to differentiation into goblet cells, which secret mucous (in particular, they express *Muc2*, which had the largest ordering coefficient identified by `psupertime`).

An alternative approach to analysing unlabelled data is to apply unsupervised pseudotime methods, and evaluate the trajectories and co-varying genes they identify. This approach may capture some of the trajectories of interest to users, but users cannot specify exactly which sequence they wish to explore. `psupertime` therefore provides a method for fast, targeted exploration of unlabelled data.

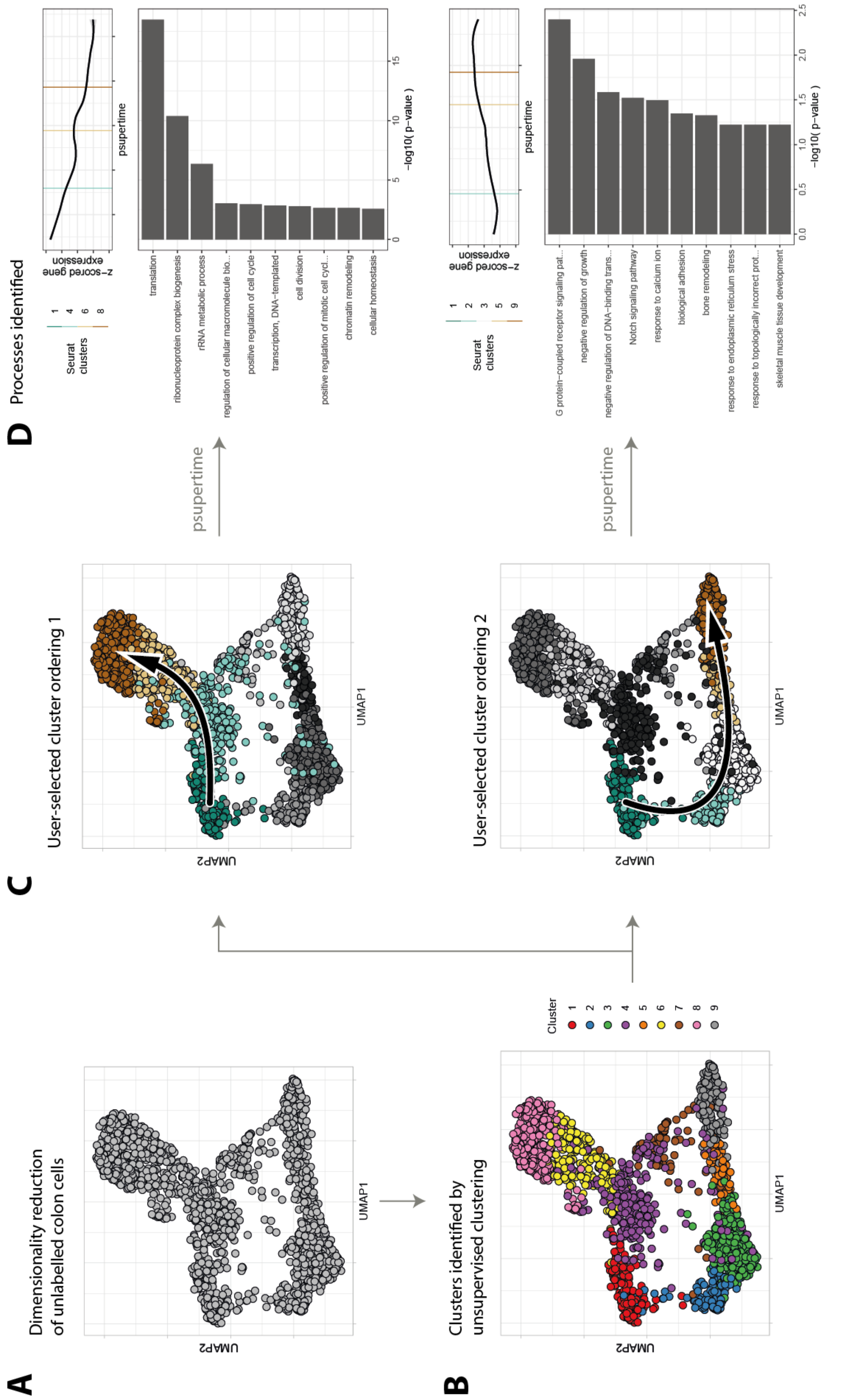

Supp Fig 14. **`psupertime`** used for exploratory data analysis

A Dimensionality reduction (`UMAP` (McInnes and Healy, 2018)) of 1894 colon cells (Herring et al., 2017). B Unsupervised clustering (via R package `Seurat` (Butler et al., 2018)) identifies 9 clusters within the sample. C Users can select cluster sequences they wish to investigate; two are shown here, which may correspond to development from stem cells into distinct mature celltypes. Arrows indicate the selected sequence. D Geneset enrichment of clustered gene profiles identifies biological processes associated with the sequence. Hierarchical clustering identified 5 gene clusters; clusters shown here are those with highest positive correlation with learned pseudotime. GO terms shown correspond to the smallest 10 p-values, subject to $p < 10\%$ and at least 5 annotated genes.

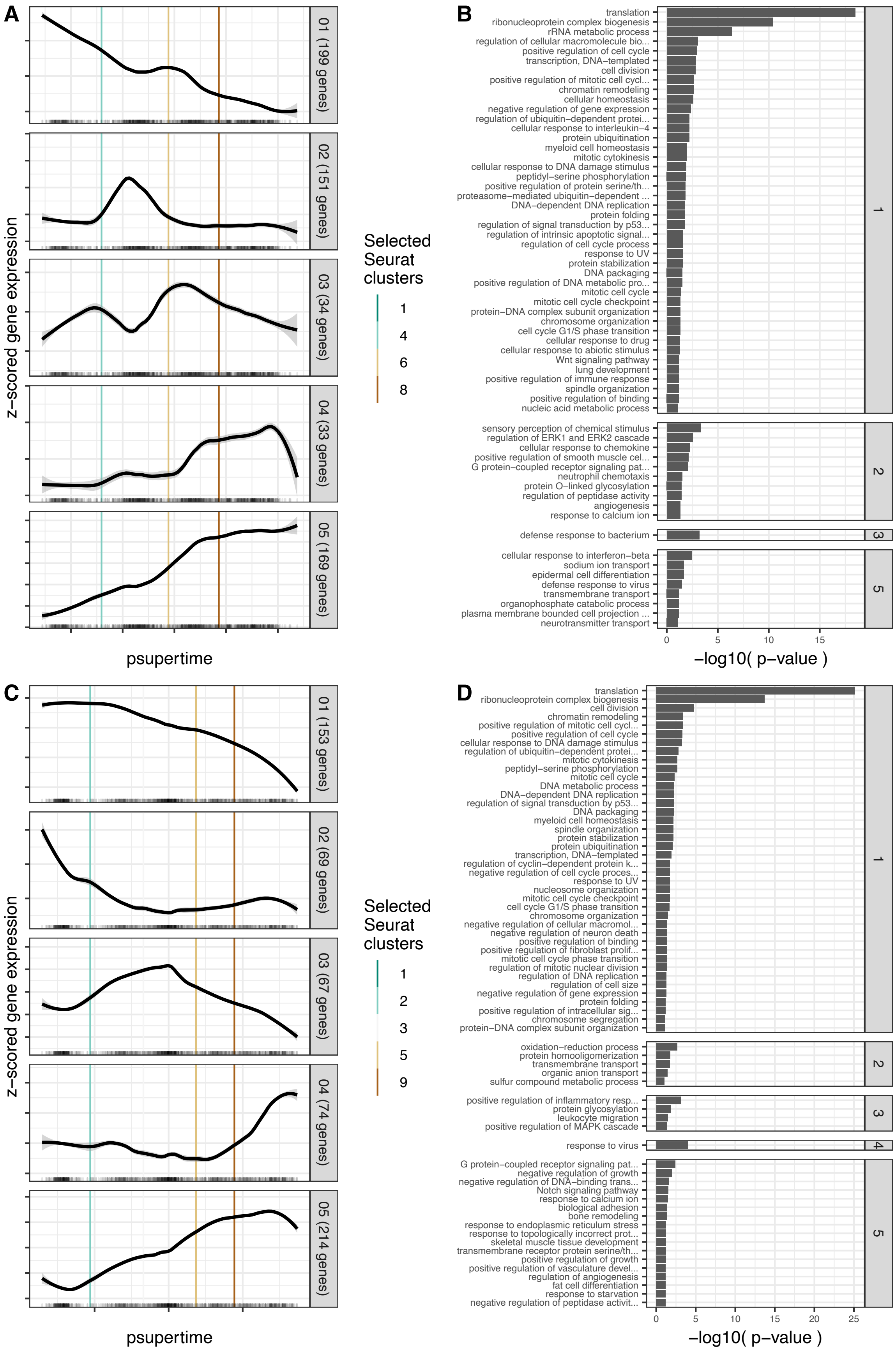

Supp Fig 15. Biological processes associated with gene profiles identified by **psupertime**. Data comprises 1894 cells from colon (Herring et al., 2017). psupertime was applied to two different user-selected cluster sequences. For each sequence, all genes selected for training were clustered into five clusters, and geneset enrichment analysis was used to identify biological processes distinctive of each cluster relative to the other four. See subsection 2.9 for details. A Clusters identified for cluster sequence 1468, ordered by correlation between mean profile and pseudotime. B Biological process GO terms identified as enriched in each cluster, relative to the remaining clusters; all GO terms shown have both Fisher exact p-value $< 10\%$ and at least 5 genes in the cluster annotated. C, D As for A,B but for cluster sequence 12359.

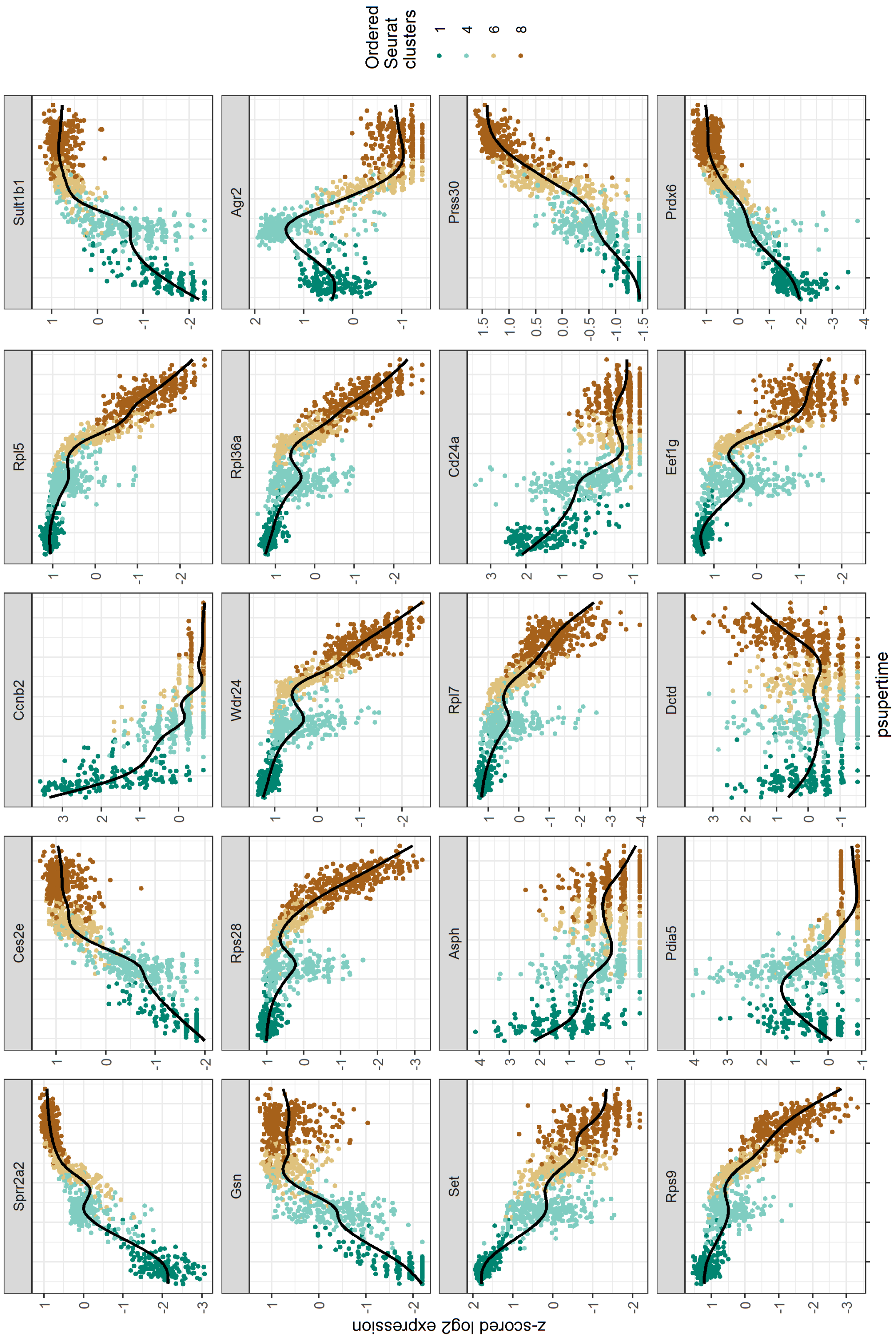

Supp Fig 16. Profiles of top genes identified by **`psupertime`** for user-selected cluster sequence 1. 20 genes with highest absolute coefficients, plotted against `psupertime` pseudotime. x-axis is the values from projections of each cell by `psupertime`. y-axis is smoothed, z-scored log pseudocounts for each cell. Colours indicate ordered labels. Black line is smoothed curve as fit by `geom_smooth` in the R package `ggplot2` (Wickham, 2016).

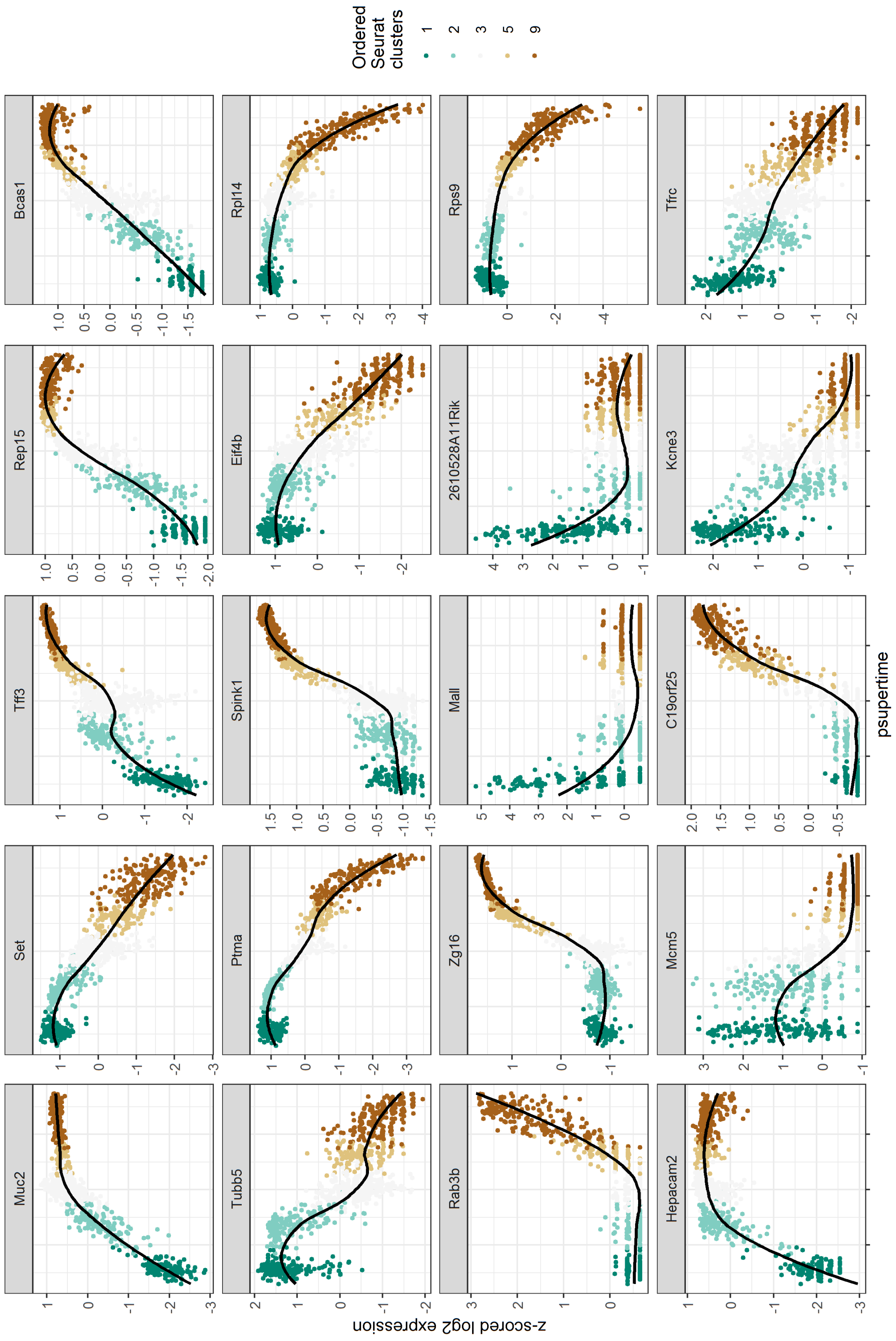

Supp Fig 17. Profiles of top genes identified by **psupertime** for user-selected cluster sequence 2. 20 genes with highest absolute coefficients, plotted against `psupertime` pseudotime. x-axis is the values from projections of each cell by `psupertime`. y-axis is smoothed, z-scored log pseudocounts for each cell. Colours indicate ordered labels. Black line is smoothed curve as fit by `geom_smooth` in the R package `ggplot2` (Wickham, 2016).

Supplementary Results 4: **psupertime** as a feature extraction method for visualization and further analysis

The set of genes reported by psupertime correspond to a subset of genes which is relevant to the ordered sequence of labels. This set of genes can therefore be used for feature selection, for example as input to dimensionality reduction algorithms, resulting in a low-dimensional embedding based only on genes specific to the biological process in question.

Restricting to the genes identified as relevant by psupertime results in an improvement in the results of dimensionality reduction algorithms (PCA and UMAP (McInnes and Healy, 2018)), with respect to continuous ordering of the sequential labels (Supp Fig 18). With the exception of the human germline data, all embeddings based on the psupertime genes consist of a smaller number of distinct cell clusters than for the highly varying genes (HVG). The recapitulation of the sequential ordering is also often improved. This is clearest in the cases where the methods already at least partly recapitulated the ordering even with the highly variable genes. Here, restriction to the genes identified by psupertime improves the ordering further; for example, applied to the MEF to neurons dataset (Treutlein *et al.*, 2016), selecting this subset of genes results in the 20 day old cells being placed in the correct ordering in the PCA plot. Overall, the genes identified by psupertime result in embeddings which better reflect the sequential labels, and fewer discontinuities between cells with similar labels.

In principle this feature selection would allow for further analysis of such processes, such as clustering of the cells, or identifying sets of genes showing similar expression profiles. We expect psupertime to improve the performance of such methods by excluding genes which are not relevant to the labels.

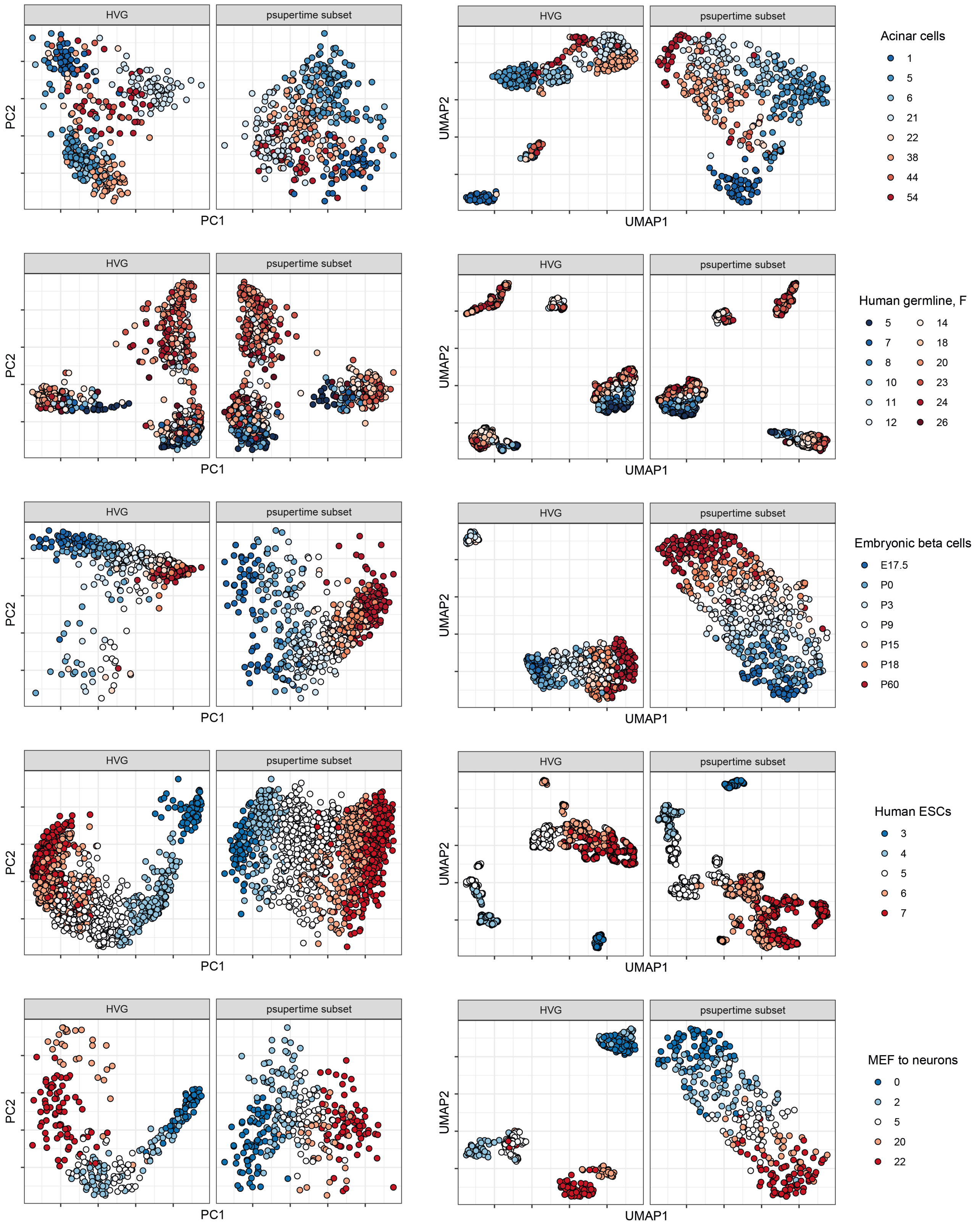

Supp Fig 18. Dimensionality reduction comparing highly variable genes and genes identified by **psupertime** as input. Rows correspond to datasets detailed in Table 1. First pair of columns shows first two principal components; second pair shows projection with `UMAP`, using default parameters (McInnes and Healy, 2018). In both pairs of columns, the left uses all highly variable genes ('HVG') as input, and the right uses only genes identified by `psupertime` as input ('psupertime subset').

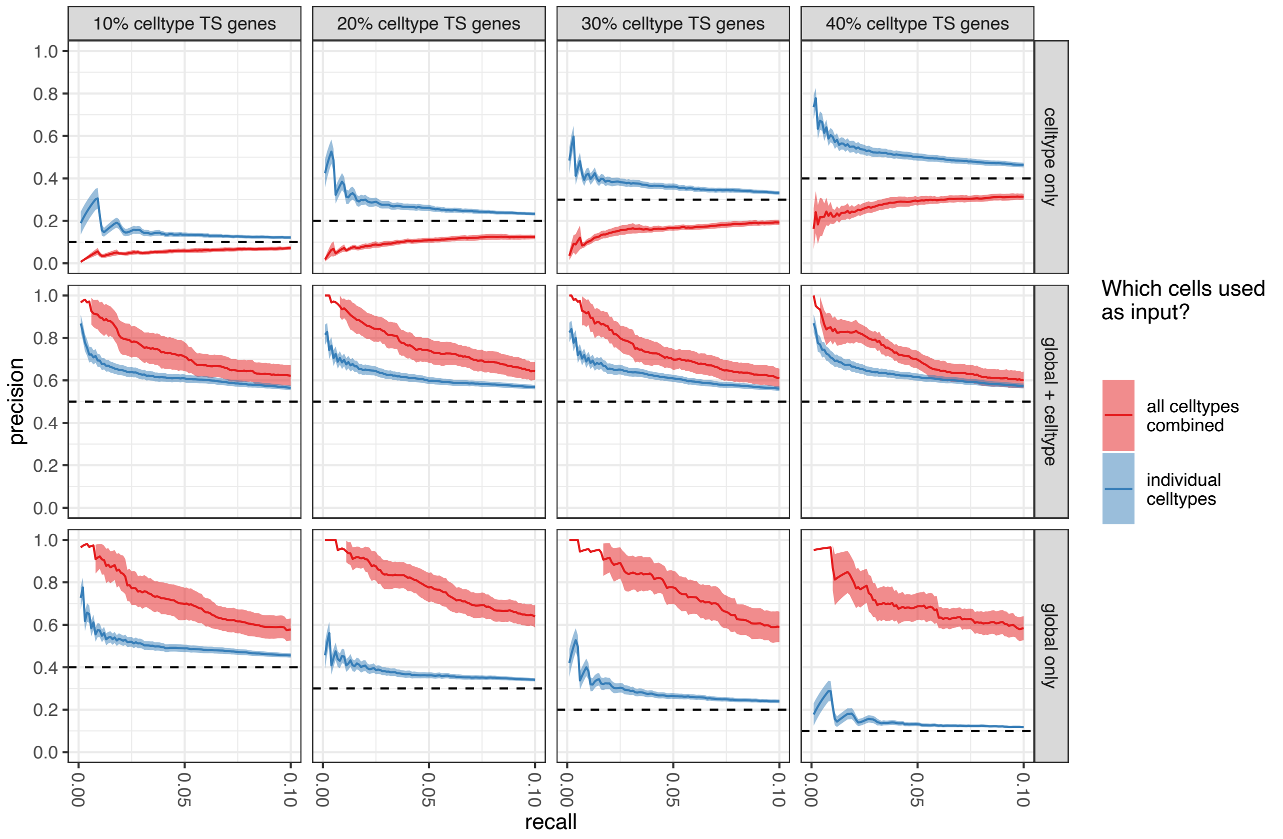

Supp Fig 19. Performance of **psupertime** on simulated datasets composed of multiple celltypes. Precision-recall curves based on identification by `psupertime` of global and celltype-specific time-series genes via absolute coefficient values. Line and area show mean and $\pm$ 2 standard error respectively, over 20 simulations with 8 simulated celltypes each. Recall is limited to range 0% to 10%, to test identification of small, relevant subset. Columns correspond to simulations with different proportions of celltype-specific vs global time-series genes; rows indicate identification of different sets of genes selected for identification. Black dashed line shows proportion of genes which are true, i.e. rate of success which would be achieved by random guessing. All simulations included 30% batch-effect genes which are sample-specific, and 20% genes with constant mean expression.

Supplementary Results 5: Application of **psupertime** to datasets comprised of multiple cell types and branches

Many single cell RNA-seq experiments are comprised of samples of multiple celltypes mixed together. This mixing of celltypes could in principle make it more difficult for `psupertime` to identify genes that vary with time: where the same gene has different profiles over time across celltypes, this signal will be made less clear by averaging over all celltypes. We sought to test this both by simulating data from multiple celltypes, and by application of `psupertime` to multiple distinct branches from the same biological dataset.

We simulated time-series single cell RNA-seq data for multiple celltypes with different response profiles within each celltype as described in subsection 2.6. After simulating count data, we applied `psupertime` to each celltype individually, and to all celltypes grouped together. We could then use the coefficients identified by each fitted `psupertime` to determine whether or not the global and celltype-specific genes were successfully identified.

We found that `psupertime` was consistently able to identify time-series genes, although the genes which were identified varied depending on whether it was applied to individual celltypes or to all celltypes together (Supp Fig 19). Applied to all celltypes, `psupertime` shows high precision in identification of genes which vary over time across all celltypes. Conversely, it performs less well in identifying genes whose time-series profiles differ between celltypes; this is expected behaviour. Application of `psupertime` to individual celltypes shows that `psupertime` is able to identify genes which vary with time in a celltype-specific way, and also globally varying genes (although to a lesser extent). This suggests that where a sample is known to be composed of multiple celltypes (or equivalently, branches), users should apply `psupertime` to both the whole dataset, and individually to each celltype (after identifying them via unsupervised techniques; see **??**). This allows users to identify both globally time-varying genes, and genes whose time-series variation is specific to individual celltypes.

Having shown that psupertime is able to identify genes which vary both globally and in a celltype-specific manner, we applied it to a dataset comprising multiple distinct trajectories leading to different cell fates. Specifically, we analysed data from mouse embryonic fibroblast cells (MEFs) reprogrammed to induced pluripotent stem cells (iPSCs), which also resulted in production of stromal cells (Schiebinger *et al.*, 2019). After pre-processing, we used dimensionality reduction (McInnes and Healy, 2018) combined with unsupervised clustering (Butler *et al.*, 2018) to identify two clear branches: one branch corresponding to reprogramming from MEFs to iPSCs, and another to reprogramming from MEFs to stromal cells (Supp Fig 20).

We applied psupertime three times: to the entire dataset; to cells with labels days 0 to 9 plus 5 clusters comprising the iPSC branch; to cells with labels days 0 to 9 plus 2 clusters comprising the stromal branch. In each case, we trained psupertime using the experimental days as labels. psupertime identified relevant genes for the global process (e.g. *Dppa5a* (Lee *et al.*, 2014)), for reprogramming to iPSCs (e.g. *Cd24a* (Shakiba *et al.*, 2015)) and for reprogramming to non-pluripotent cells (e.g. *Xist* (Minkovsky *et al.*, 2012)) (Supp Fig 21).

Applied globally, psupertime is able to identify genes with relevant profiles even where the signal is diluted by the presence of branches showing different profiles. Supp Fig 22A shows the 10 genes with highest absolute coefficients for psupertime applied to the whole Schiebinger *et al.* dataset. For several genes, later labels show bimodal expression (e.g. *Dppa5a*, *Igfbp2*, *Bex1*). Here, the global expression profile is a mixture of profiles from two branches with different profiles. Supp Fig 22B shows the same genes, but the cells and corresponding pseudotime values from psupertime applied to the iPSC branch only. Here, the expression profiles are unimodal and as a result show lower variance. Despite the high variance in the global case, psupertime successfully identified relevant genes. This indicates that even where the branching or celltype status of a dataset is unclear (and the user therefore only does analysis at a global level), psupertime is still able to identify genes which are relevant to the process of interest.

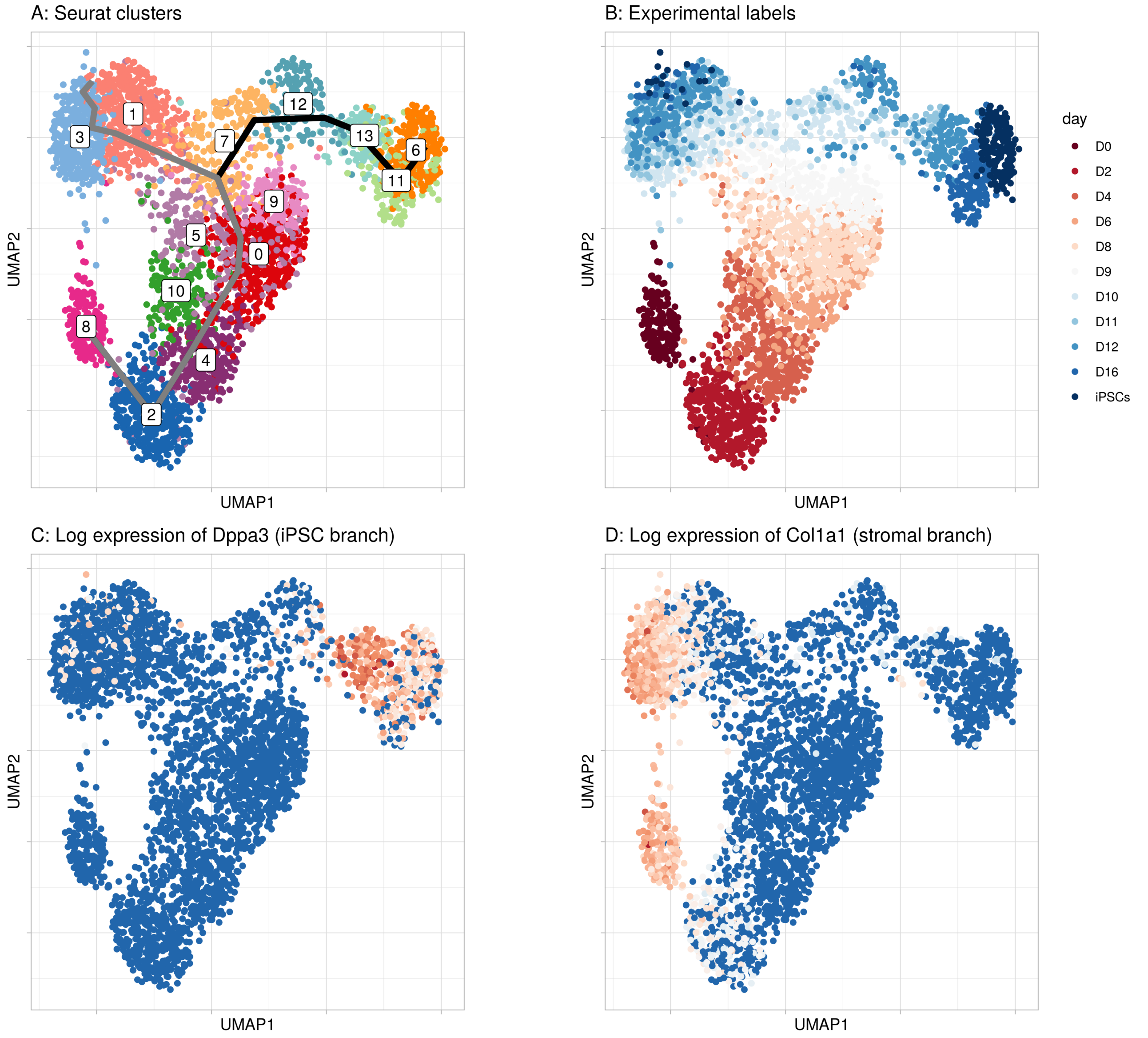

Supp Fig 20. Identification of branches within iPSC data. Data comprises 3600 cells, with labels D0 to D8 under DOX condition and labels D9 to iPSCs under 2i condition (Schiebinger et al., 2019). A Definition of branches by selection clusters which corresponded to the clear branching structure: 7, 12, 13, 11, 6 for iPSCs; 1, 3 for stromal. Days D0-D9 were defined as common to both branches. Grey and black lines show the medians of the experimental labels for each branch. B Experimental labels shown over `UMAP` layout. C Log expression of iPSC marker gene over `UMAP` layout. D Log expression of stromal marker gene over `UMAP` layout.

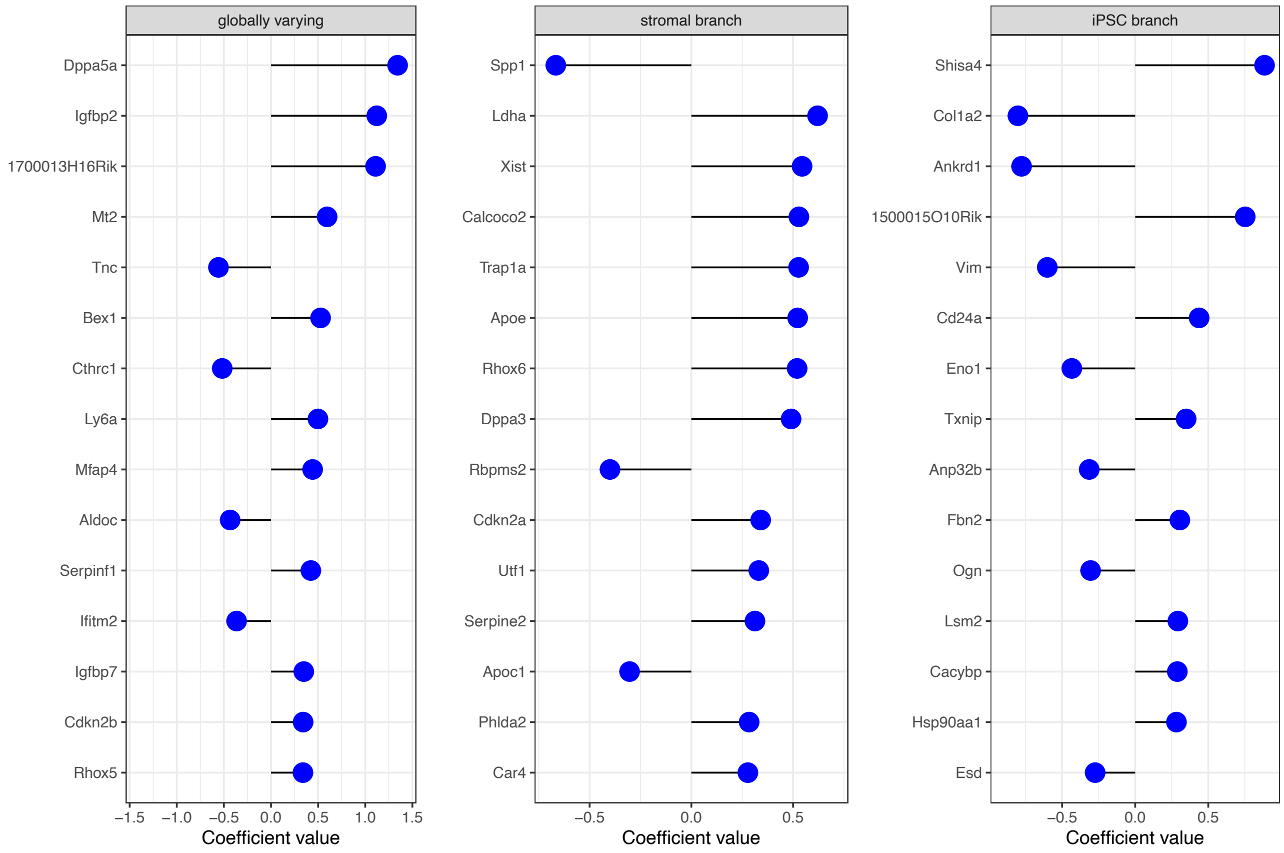

Supp Fig 21. Identification of genes co-varying with days across whole dataset and individual branches. `psupertime` was applied to three different sets of cells: the whole dataset; days D0-D9 plus the iPSC branch; and days D0-D9 plus the stromal branch. In each case, the labelled days were used as training labels. The genes shown are the 15 genes with largest absolute coefficient, subject to constraints on branch expression. Genes identified as unique to a branch must have absolute coefficient above 0.05 for this branch, and below 0.05 in the psupertime for the other branch. Globally varying genes have no constraints.

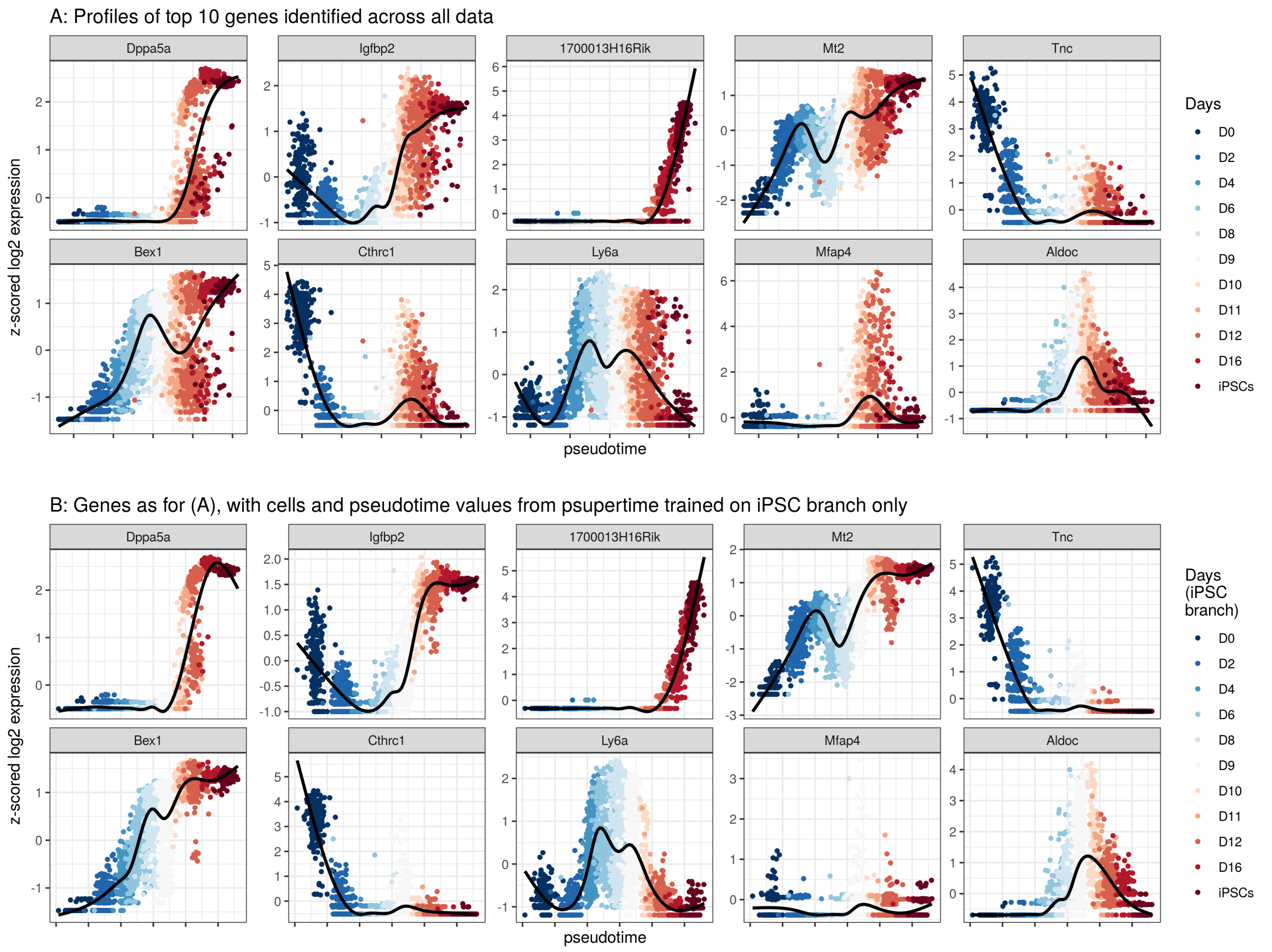

Supp Fig 22. Profiles of genes identified in iPSC dataset. A `psupertime` was applied to the whole dataset, using the labelled days as training labels. 10 genes with highest absolute coefficients, plotted against `psupertime` pseudotime. x-axis is the values from projections of each cell by `psupertime`. y-axis is smoothed, z-scored log pseudocounts for each cell. Colours indicate ordered labels. Black line is smoothed curve as fit by `geom_smooth` in the R package `ggplot2` (Wickham, 2016). B Genes as for (A), but with cells and pseudotime values derived from `psupertime` applied to iPSC branch only.

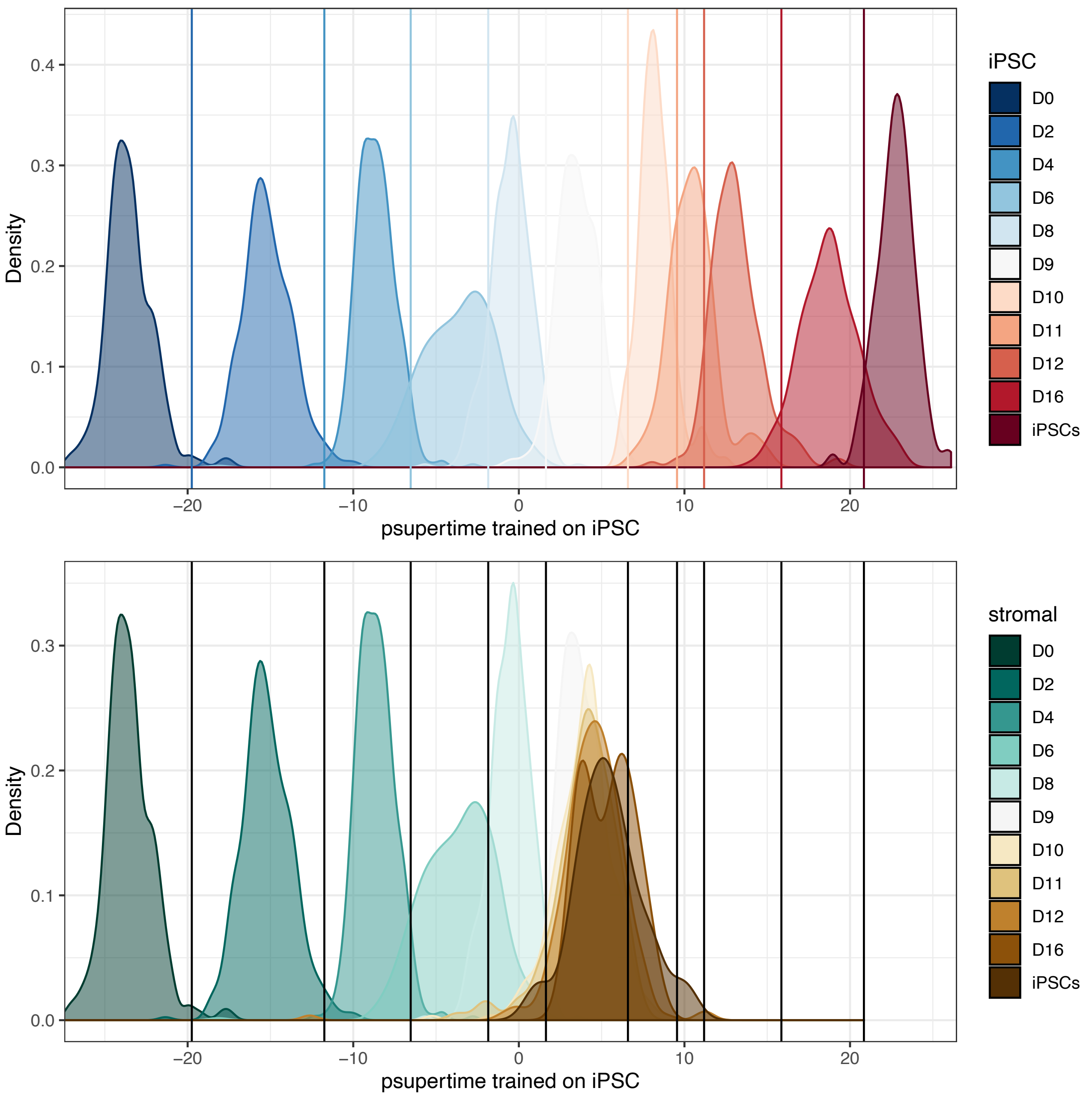

Supp Fig 23. **`psupertime`** trained on iPSC branch, used to predict pseudotime values for stromal branch. Data comprises 3600 cells, with labels D0 to D8 under DOX condition and labels D9 to iPSCs under 2i condition (Schiebinger et al., 2019). `psupertime` was trained on branch 1 Supp Fig 20. The first row shows the distribution of the labels for these cells, and the corresponding pseudotime values from `psupertime`. The second row shows the results of applying this `psupertime` to cells from branch 2. The labels are the true labels from this condition, and the branch 1-trained `psupertime` was used to predict their x-axis values. For the cells over days D0 to D8, the cells used are identical, resulting in identical distributions over the pseudotime. On both plots, the vertical lines indicate thresholds between the labels for branch 1.

This analysis demonstrates a further capability of `psupertime`: once it is trained, it can then be used to predict labels for new data with different or unknown labels (e.g. the branches identified in the iPSC data). We used the `psupertime` trained on cells committing to the iPSC fate to predict the pseudotime values for cells committing to the stromal fate (Supp Fig 23). This indicates that the committed stromal cells are only equivalent to day 9 with respect to the reprogramming process for iPSCs (Supp Fig 24). `psupertime` can thus be used to assess cells obtained under one process with respect to another.

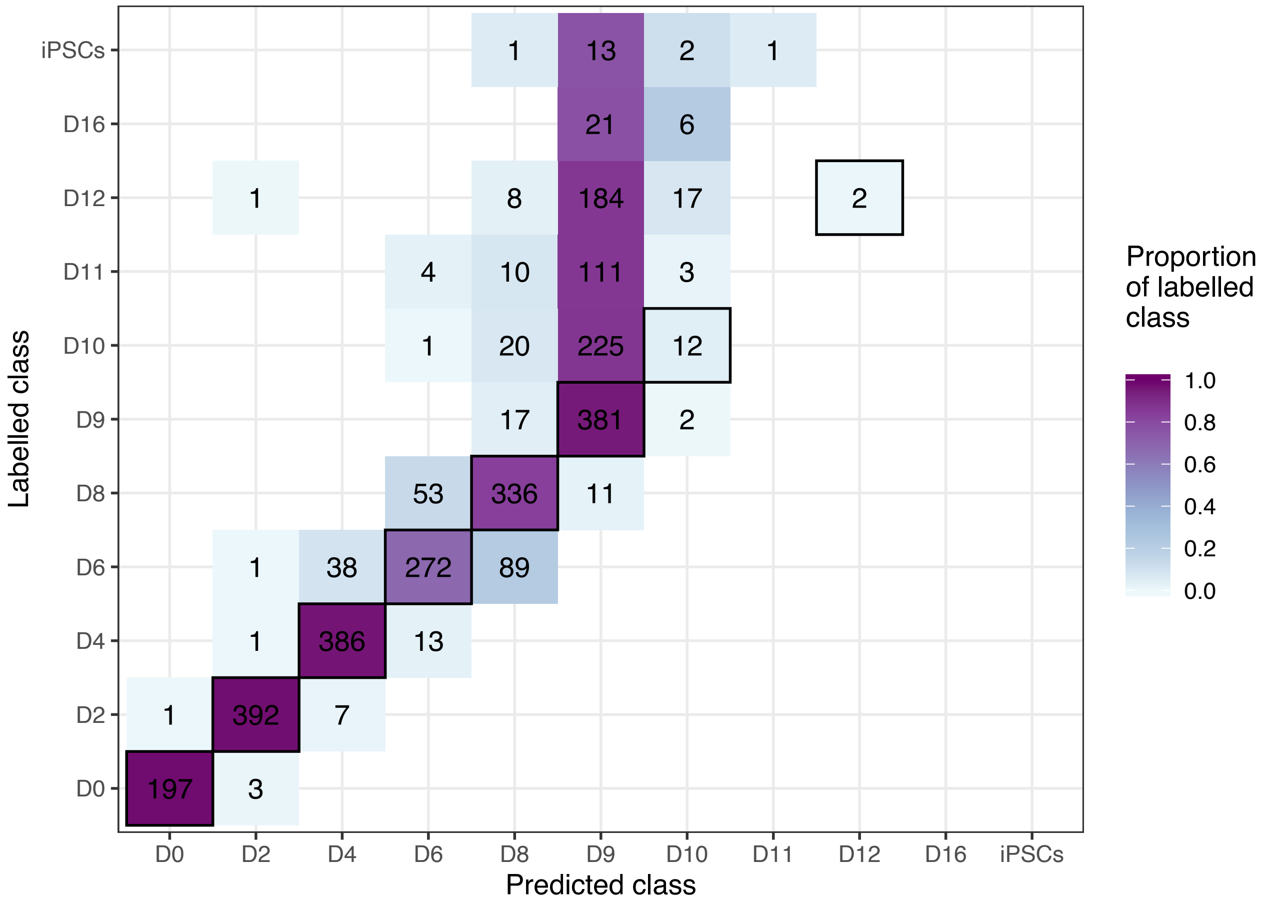

Supp Fig 24. **`psupertime`** trained on iPSC branch, used to predict labels for stromal branch. Data and application of `psupertime` as for Supp Fig 23. The rows show the true labels of cells from the stromal branch. The columns show the predicted labels from the `psupertime` trained on the iPSC branch. Numbers correspond to number of cells with this combination of predicted and true labels.

Supplementary Results 6: Potential applications and developments of **psupertime**

We have shown that where sequential labels are available, psupertime is able to to identify genes whose expression profiles correspond to this order. It does this even in the presence of substantial unrelated variation, and does so better than benchmark unsupervised methods. psupertime is conceptually simple, and its simplicity allows for several avenues for future development.

We performed comparisons between psupertime and other unsupervised methods on the basis of ability to recapitulate the ordering of the known labels. Cellular responses are heterogeneous, however, meaning that this known label sequences are imperfect labels of a cell’s progress along a given process. Unsupervised pseudotime techniques explore this heterogeneity by basing their orderings solely on similarities between cells. This suggests a complementary approach between psupertime and unsupervised methods: psupertime can be used to identify the genes and ordering correlating with the label sequence, and this can be compared with results from unsupervised approaches to quantify the extent of heterogeneity.

While the pseudotime identified by psupertime may have a non-linear relationship with the condition labels (for example, in the case where a gene has zero expression for early labels, and a constant higher level of expression for later labels), it is a linear function of the gene expression values. Non-linear implementations of psupertime (e.g. as a neural network, or via non-linear regression such as MARS (Friedman, 1991)) would allow for pseudotimes which were non-linear, non-monotonic functions of the genes, which would in particular permit the identification of genes showing transient expression.

The design of many single cell studies results in sequential groups of cells (see for example reviews on development (Kumar *et al.*, 2017) and aging (Song *et al.*, 2018)). psupertime has been developed for single cell RNA-seq data, however it could in principle also be applied to other single cell data such as mass cytometry (Bendall *et al.*, 2011). We have shown good performance for psupertime for single cell RNA-seq data, even though we would not *a priori* expect that a biological process would correspond to a linear combination of gene expressions. This may be the result of the high dimensionality of the dataset, which provides a large set of features with which to approximate a non-linear process. Data derived from mass cytometry is lower-dimensional, and therefore has lower flexibility of marker choice. Here, a non-linear model may be necessary to obtain good performance.

As a classifier, psupertime can be first trained on cells with one set of condition labels, then used to project new cells onto the associated process. We showed this by applying psupertime to a time course of iPSCs allowed to differentiate, and used this to classify iPSCs kept in pluripotency-maintaining serum (Supp Fig 23). This illustrates potential uses for psupertime to compare processes. For example, psupertime could be trained on a time series of stimulated cells and used to test the effects of inhibitors: the locations of the inhibited cells, projected onto the pseudotime corresponding to the *uninhibited* process, would indicate the timepoint at which the inhibitor acted.

psupertime uses L1 regularization to obtain a small set of reported genes. However, this may result in exclusion of other relevant genes: where there are multiple highly correlated genes that are predictive of the sequential labels, L1 regularization will tend to result in only one of these genes being reported, and produce give zero coefficients for other correlated genes . This issue can be addressed by calculating the psupertime ordering, and reviewing all genes that have high correlations with the genes identified by psupertime. Alternatively, a trivial extension to psupertime would allow training with a combination of L1 and L2 penalties (the *elastic net*), resulting in a compromise between sparsity and prediction performance.

psupertime is applicable to many of the increasing number of single cell RNA-seq studies being generated. It shows consistently better performance than benchmark methods, due to use of sequential labels as input. The conceptual novelty of identifying genes via ordinal logistic regression both permits genes relevant to processes annotated with sequential labels to be identified, and suggests new ways of using these labels to understand the genes involved in such processes.
